# Optimization and prediction of the electron–nuclear dipolar and scalar interaction in ^1^H and ^13^C liquid state dynamic nuclear polarization†
†Electronic supplementary information (ESI) available: Additional mathematical derivations, DNP experimental setup and procedures for DNP parameter determination, *a*_FC_*via* DFT modeling and considered configurations for investigated systems, additional ^13^C DNP enhancement results for above mentioned systems, and full coordinates for investigated configurations. See DOI: 10.1039/c5sc02499d


**DOI:** 10.1039/c5sc02499d

**Published:** 2015-07-29

**Authors:** X. Wang, W. C. Isley III, S. I. Salido, Z. Sun, L. Song, K. H. Tsai, C. J. Cramer, H. C. Dorn

**Affiliations:** a Department of Chemistry , Virginia Tech , Blacksburg , Virginia 24061 , USA . Email: hdorn@vt.edu; b Department of Chemistry and Minnesota Supercomputing Institute , University of Minnesota , Minneapolis , Minnesota 55455-0431 , USA . Email: isley009@umn.edu

## Abstract

During the last 10–15 years, dynamic nuclear polarization (DNP) has evolved as a powerful tool for hyperpolarization of NMR and MRI nuclides. However, it is not as well appreciated that solution-state dynamic nuclear polarization is a powerful approach to study intermolecular interactions in solution. For solutions and fluids, the ^1^H nuclide is usually dominated by an Overhauser dipolar enhancement and can be significantly increased by decreasing the correlation time (*τ*_c_) of the substrate/nitroxide interaction by utilizing supercritical fluids (SF CO_2_). For molecules containing the ubiquitous ^13^C nuclide, the Overhauser enhancement is usually a profile of both scalar and dipolar interactions. For carbon atoms without an attached hydrogen, a dipolar enhancement usually dominates as we illustrate for sp^2^ hybridized carbons in the fullerenes, C_60_ and C_70_. However, the scalar interaction is dependent on a Fermi contact interaction which does not have the magnetic field dependence inherent in the dipolar interaction. For a comprehensive range of molecular systems we show that molecules that exhibit weakly acidic complexation interaction(s) with nitroxides provide corresponding large scalar enhancements. For the first time, we report that sp hybridized (H–C) alkyne systems, for example, the phenylacetylene–nitroxide system exhibit very large scalar dominated enhancements. Finally, we demonstrate for a wide range of molecular systems that the Fermi contact interaction can be computationally predicted *via* electron–nuclear hyperfine coupling and correlated with experimental ^13^C DNP enhancements.

## Abstract 2

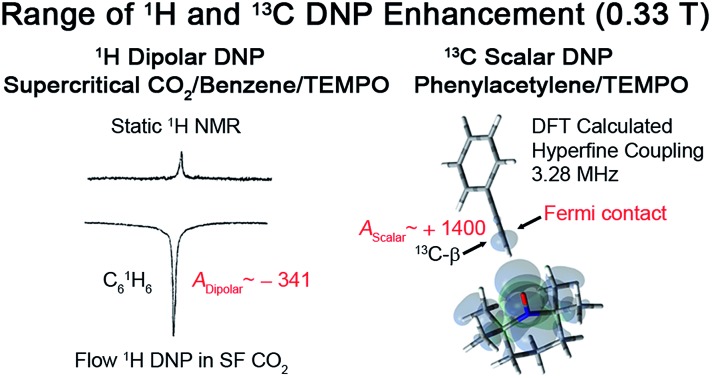
Solution-state dynamic nuclear polarization (DNP) is a powerful tool for hyperpolarization and the study of intermolecular interactions in solution.

## Introduction

In dynamic nuclear polarization, the electron–nucleus dipolar and scalar couplings can be described by the Hamiltonian,

1 *Ĥ* = *hŝDÎ* + *hÎAŝ*

where *h* is the Planck constant, *Î* is the nuclear spin angular momentum operator, *ŝ* is the electronic spin angular momentum operator, *D* is the dipolar coupling tensor, and *A* is the hyperfine coupling tensor (typically in units of MHz). One can compute the dipolar coupling tensor *D*; however, knowledge of the electron–nucleus dipole–dipole correlation function is required to determine the enhancement factor.^1^ Computation of this dipole–dipole correlation function can be achieved with a molecular dynamics simulation. However, such simulations are typically limited to select parameterized systems, and are very computationally intensive.^1^–^3^

**Fig. 1:**
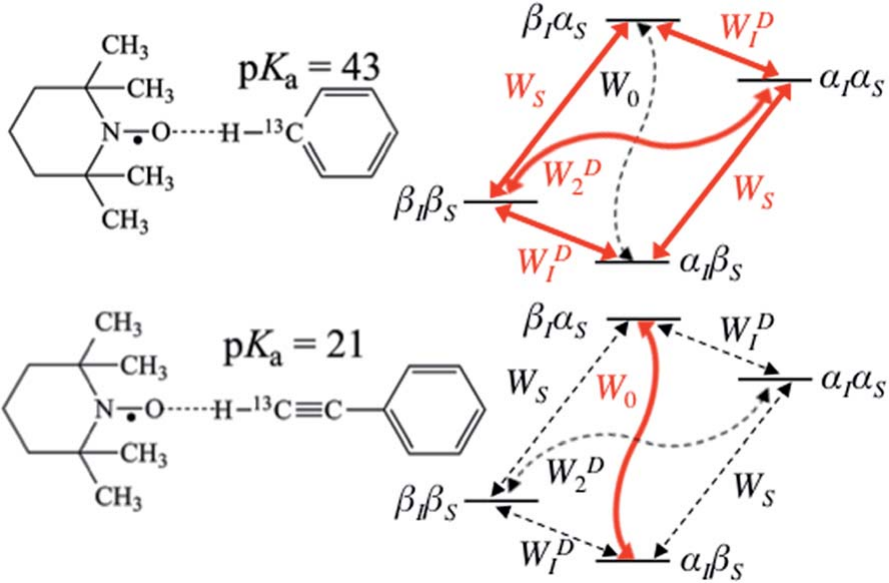
Schematic diagram of energy level and transition probabilities for electron–nuclear (^13^C) spin systems for benzene/TEMPO (above) and phenylacetylene/TEMPO (below). α_S_ and β_S_ are the spin states of the unpaired nitroxide electron spin, and α_I_ and β_I_ are for nuclear spins. *W*_S_ represents the electron relaxation; *W*_0_, *W*D1 and *W*D2 are zero, single and double quantum transitions, respectively. *W*_0_ is equal to the sum of *W*Sc0 and *W*D0 in eqn (3) and (4), which are transition rates of scalar and dipolar relaxation, respectively. ^13^C DNP enhancement of benzene is mainly due to dipolar relaxation, while scalar relaxation is the dominant mechanism for the β carbon of phenylacetylene.

**Fig. 2:**
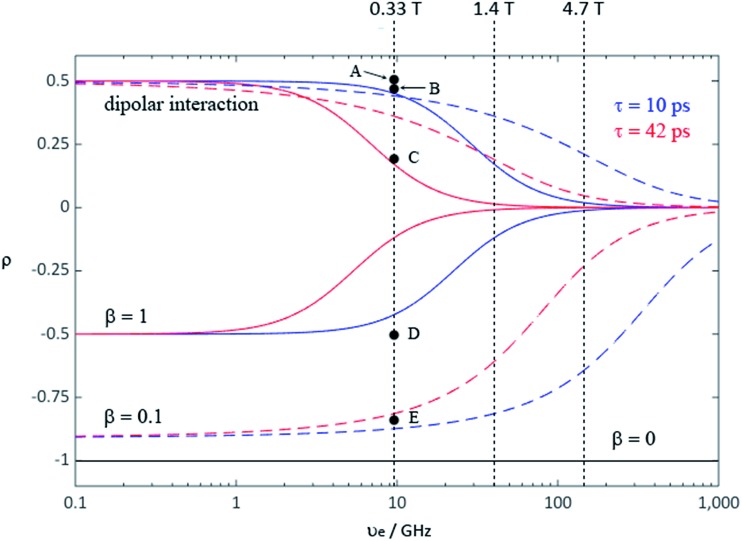
Coupling factor (*ρ*) of the Overhauser effect enhancement depends on the electron Larmor frequency for rotational (solid line) and translational (dash line) motion with correlation time 10 ps (blue) and 42 ps (red); mixed with scalar enhancement of *β* = 1 (blue solid for *τ*_c_ = 10 ps and red solid for *τ*_c_ = 42 ps), and *β* = 0.1 (blue dash for *τ*_c_ = 10 ps and red dash for *τ*_c_ = 42 ps), where *β* values define different contributions from dipolar relaxation competing with pure scalar relaxation.^34^ Pure scalar enhancement (black solid line) is independent of magnetic field strength. Points A–E represent coupling factor values calculated from extrapolated experimental LLIT DNP enhancements polarized at 0.33 T: (A) ^1^H of 9% benzene/0.001 M TEMPO/SF CO_2_,^43^ (B) ^1^H of chloroform/0.0015 M TEMPO^44^, (C) ^1^H of 9% benzene/0.01 M TEMPO/C_6_D_6_,^43^ (D) ^13^C-β of phenylacetylene/0.01 M TEMPO^45^, and (E) ^13^C of chloroform/0.0052 M TEMPO.^44^ The relative standard deviation for *ρ* is about 15%. The two correlation times were estimated from ^1^H DNP coupling factors of benzene in SF CO_2_ and normal solvent by a rotational diffusion modulated dipolar interaction mechanism (see ESI† for derivation).

During the last two decades, significant advances have been made in the field of dynamic nuclear polarization (DNP).^4^,^5^ For liquid-state DNP, there has been a resurgence of interest in ameliorating liquid-state high magnetic field DNP enhancements.^6^–^10^ Liquid state Overhauser effect DNP has been applied for signal enhancement of flow imaging^11^–^14^ as well as the characterization of local water dynamics in biological systems.^15^–^21^ More recently, ^1^H liquid state DNP measurements of water^22^–^26^ and small organic molecules^27^–^29^ at higher magnetic fields have been reported. However, liquid state DNP experiments also provide an excellent approach for understanding and predicting intermolecular solution interactions. The drawbacks for obtaining significant DNP enhancements (*A*) in the liquid state are well recognized, since the time-dependent electron–nuclear interaction dominates the Overhauser effect (Oe).^30^–^35^ This can be derived from eqn (2)–(4), where *γ*_S_ and *γ*_I_ are the magnetogyric ratios for the electron and nuclear spins, respectively, and the coupling (*ρ*) and leakage (*f*) factors are defined in terms of the nuclear transition probabilities in the presence of a free radical, *W*D2, *W*D0, *W*D1, which represent dipolar relaxation, and *W*Sc0 due to scalar relaxation, as well as the transition probability in the absence of a free radical *W*_10_ (Fig. 1).^35^ For molecular liquid systems, the time-dependent dipolar interaction dominates at low magnetic fields and/or short correlation times (*τ*_c_) in the extreme narrowing limit *ω*_S_^2^*τ*_c_^2^ ≪ 1 (*ω*_S_ electron Larmor frequency). Unfortunately, at the high magnetic fields (3–12 T) commonly employed in NMR, the coupling factor approaches zero (shown in Fig. 2).
2

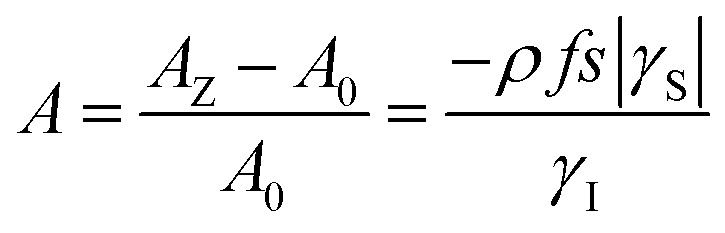



3

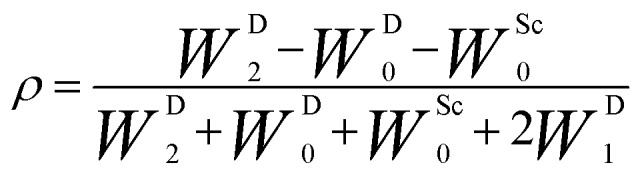



4

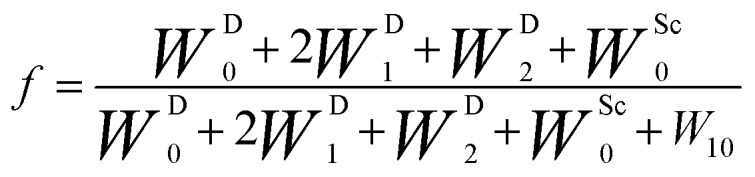




Scalar relaxation originates from the coupling of the magnetic moments of electrons and nuclei *via* the hyperfine coupling interaction.^36^ Although *A* in eqn (1) is the isotropic Fermi contact hyperfine coupling tensor, henceforth it is labelled as *a*_FC_, in order to avoid confusion with the DNP enhancement, *A vide infra*.

For hydrogen containing molecules, ^1^H DNP enhancements are usually dominated by the dipolar relaxation mechanism. If one assumes a rotationally modulated mechanism dominates the electron–nuclear dipolar interaction, the corresponding rotational correlation time (*τ*_r_) can be estimated^34^,^35^,^37^ (see ESI† for detailed derivation). The corresponding DNP coupling factor modulated by exclusive rotational diffusion gives the magnetic field dependence shown in Fig. 2. More recently, it has been reported that translational diffusion predominantly modulates the dipolar interaction for water^6^,^38^,^39^ and some small organic molecular systems^29^,^40^ with organic radicals. Using the same correlation times, the field dependence of the DNP coupling factor modulated by translational diffusion^33^,^35^,^41^ is also represented by coloured dashed lines in Fig. 2. One notable exception for the ^1^H nuclide is a nitroxide–trifluoroacetic acid system reported by Bates where the scalar interaction dominates the ^1^H DNP enhancement.^42^

On the other hand, for the ^13^C nuclide, the coupling interaction can be dominated by a strong scalar interaction (*W*Sc0 ≫ *W*D2, *W*D1, *W*D0) or a modest dipolar interaction (*W*Sc0 ≪ *W*D2, *W*D1, *W*D0), or by a mixture of scalar and dipolar interactions. As illustrated in Fig. 1, it is well recognized that molecules with a (C–H) group can exhibit weak hydrogen bonding with nitroxides and show modest to large scalar enhancements.^35^,^44^,^46^–^49^ Griffin and coworkers have pointed out the importance of the scalar interaction for certain other nuclides (^19^F, ^15^N, ^31^P) besides ^13^C for high-field liquid-state DNP studies.^50^ Unfortunately, it is difficult to estimate the corresponding scalar correlation time *τ*_sc_. For the case of acetonitrile/TEMPO, we previously reported ^13^C DNP *τ*_sc_ values 2–3 times longer than the dipolar correlation time (*τ*_r_).^49^

As first reported by our laboratory, the solid–liquid intermolecular transfer (SLIT) DNP experiment has certain advantages when the radical is not present in the solution.^51^,^52^ In the SLIT DNP experiment, ^13^C contact shifts and spectral line broadening are avoided in the high field NMR detection magnet. The SLIT approach also has the advantage of improved low to high magnetic field transfer efficiencies allowing the transfer of radical free hyperpolarized metabolites for biological uses.^53^,^54^ The use of immobilized radicals for DNP approaches has recently been reconsidered and utilized to generate hyperpolarized water for clinical MRI applications.^11^–^13^,^55^,^56^ The efficiency of interactions between the radical and nuclei in immobilized radical flow methods can be in fact improved through better design of immobilizing methods,^11^,^57^ as well as the optimum synthesis of new radicals.^58^–^60^

In the current study, we demonstrate that ^1^H LLIT (liquid–liquid intermolecular transfer) and ^1^H SLIT DNP with solutes dissolved in supercritical fluid (SF) CO_2_ result in enhanced electron–nuclear dipolar interactions and exhibit significant Oe DNP enhancements as a result of reduced correlation times. This is the first report of SF DNP with supercritical CO_2_ except for the earlier ^1^H DNP enhancement of supercritical ethylene by Wind and coworkers.^61^ It is well recognized that solutes dissolved in supercritical fluids exhibit significantly shorter molecular correlation times.^62^

For the case of the ^13^C nuclide, we present dipolar dominated ^13^C DNP enhancements for the fullerene C_70_, with a trend attributable to the difference in the intermolecular distance for electron–nuclear interaction. In a large number of molecules with acidic C–H groups, significant scalar interactions and corresponding large positive enhancements are observed in the nitroxide–substrate complex. For example, we show that sp hybridized (H–C) alkyne systems, such as, the phenylacetylene–nitroxide system exhibit very large scalar dominated enhancements. It is also possible to directly compare liquid–liquid intermolecular scalar (Fermi contact) hyperfine couplings (*a*_FC_) with experimental scalar DNP data utilizing density functional theory (DFT) modeling.^49^ We will subsequently demonstrate that computational predictions of the scalar hyperfine coupling constants (HFCs) across a wide set of nitroxide–substrate systems correlate strongly with the DNP enhancements for systems dominated by the scalar interaction.

## Experimental

### Samples

The production method used to obtain the C_60_ and C_70_ fullerenes utilized an apparatus similar to the original Krätschmer–Huffman^63^ arc-burning method. Cored rods (6 mm diameter) packed with a mixture of graphite and amorphous carbon (^13^C labelled) were arc-burned in a fullerene generator under a dynamic He atmosphere. Typical electric-arc operating production parameters consist of 20–25 V, 95–115 A, and 180–220 Torr of helium. The fullerenes were extracted by dissolution in CS_2_ immediately after collection of the soot. Following this extraction protocol, the raw extract was filtered over a plug of glass wool. The C_60_ and C_70_ samples were separated chromatographically utilizing a pentabromobenzyl column, (PBB, 25 cm × 10 mm i.d., Phenomenex Co., Torrance, California) with CS_2_ as the mobile phase. A mass spectrometer for the sample confirmed that the sample was ^13^C labelled at a level of approximately 12%. All other reagents and solvents for the LLIT DNP experiments were obtained commercially. C_6_D_6_ was purchased from the Isotic Inc, and other chemicals were purchased from Aldrich Chemical Co. and used without further purification. Sample solutions for the LLIT DNP experiments were prepared with TEMPO of varied concentrations from 0.001 to 0.14 M. The immobilized nitroxide materials used for the SLIT DNP experiments were synthesized in our lab by Rossi Gitti.^53^ The sample solutions were degassed by bubbling N_2_ gas through during DNP experiments.

### Dynamic nuclear polarization instrument

The instrument used for the LLIT DNP experiments was described previously.^44^ The flow transfer DNP apparatus for normal liquids and supercritical fluid CO_2_ is shown in Fig. 4, where SF CO_2_ can be used as the flowing solvent. For flow ^1^H DNP performed under supercritical fluid conditions, N_2_ degassed neat liquid benzene containing free radical TEMPO (0.1 M–0.01 M) originates at the HPLC pump and flows at a rate of 0.1 mL min^–1^. The typical sample volumes inside the low field microwave cavity and high field NMR detector are 160 and 60 μL, respectively, and the volume of the transfer region is 80 μL. CO_2_ is pumped from a syringe pump of a Suprex SFC/200A Multipurpose Unit and equilibrated to a flow rate of 1.01 mL min^–1^ and 165 atm. The liquid sample is introduced into the CO_2_ flow, and at a total flow rate of 1.11 mL min^–1^ the sample solution enters the SFC oven where it is heated to 40 °C. The flow rate maintains the liquid sample concentration ensuring the liquid solutes solubility and also keeps optimum condition based on transfer and residence times for the flow ^1^H DNP apparatus.

**Fig. 3:**
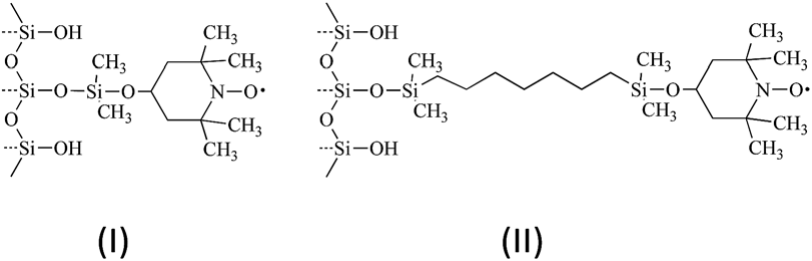
Immobilized nitroxide material I (left) and II (right) used in SLIT ^1^H DNP in normal liquids and SF CO_2_.

**Fig. 4:**
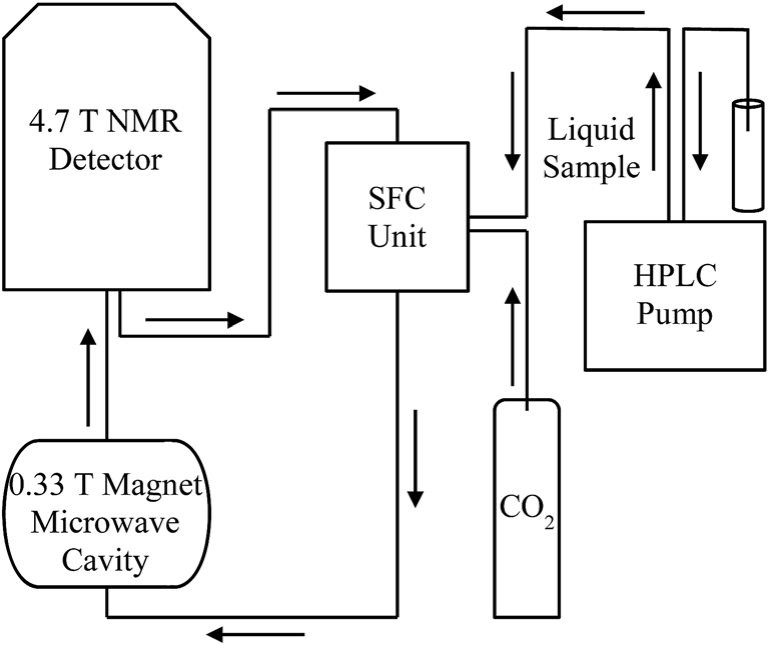
Schematic drawing of apparatus for continuous-flow HPLC-DNP-NMR coupled with SF CO_2_ (adapted from ref. 43, with the original figure shown in ESI†).

The instruments for the SLIT DNP performed in normal liquids and SF CO_2_ were reported in our previous studies.^51^–^54^ The immobilized nitroxide radical is contained in a flow cell and located in the 0.33 T low field cavity, where electron spins are polarized. The experimental procedure of SLIT ^1^H DNP is similar to that of LLIT ^1^H DNP. The rate of liquid flow containing the sample was adjusted accordingly to obtain preferred concentrations in normal liquids or SF CO_2_ as the flowing solvent.

### Determination of experimental parameters

The determination of the leakage factor *f* and the electron spin saturation factor *s* is described previously for the DNP flowing system.^44^ In ref. 44, we reported a theoretical model for determining the absolute enhancement for the low to high magnetic field transfer experiments, which is scaled according to the observed enhancement considering relaxation losses during the transfer. This model is restated in ESI.† For most systems reported in this study, the DNP data were processed based on this model, and the absolute DNP enhancements were calculated using the method we applied previously.^53^,^64^,^65^ For benzaldehyde, nitrobenzene, toluene, diphenylmethane, triphenylmethane, benzonitrile, anisole, phenylamine and acetone systems, the absolute enhancements of ^13^C LLIT DNP were obtained utilizing a ratio method^66^–^68^ using the ^13^C enhancement of cyclohexane as the reference compound, with detailed procedures illustrated in the ESI.† Enhancements of ethyl acetoacetate and diethyl malonate were determined by the ratio method using ^13^C of benzene as the reference. The DNP enhancements of C_70_ were also determined using the ratio method using C_60_ as the reference. Compared to the former model, the ratio method does not require extrapolation from observed enhancements at varied liquid flow rates and microwave powers. The applicability of the ratio method was carefully evaluated utilizing the adamantane/TEMPO system, where the DNP enhancement was validated by this method using different microwave power levels (Table S1†). A detailed discussion is included in the ESI.† However, the ratio method does lead to low-to-high magnetic field transfer efficiency errors when the substrate molecules of interest contain quaternary carbons with significantly longer *T*_1_ relaxation times. For the electron spin saturation parameter *s*, we employed relatively high radical concentrations (0.1 M and higher) for DNP experiments in normal solvents in this work, in order to saturate a single EPR line and also have leakage factors approaching unity. For the benzene/SF CO_2_ studies a low nitroxide radical concentration (0.001 M) was employed since fast Heisenberg electron–electron exchange led to a single broad EPR line for this system.

### Theory: geometry optimization, solvation, and thermochemistry

Geometry optimizations were performed for all species at the unrestricted M06-L^69^ level of density functional theory. The def2-SVP basis set^70^ was used for all atoms during optimization. The nature of stationary points was assessed in all cases by computation of analytic vibrational frequencies, which were also used to compute the molecular partition functions necessary to predict 298.15 K thermochemical quantities using the conventional ideal-gas, rigid-rotator, quantum-mechanical quasi-harmonic-oscillator^71^ approximation.^72^ Improved electronic energies were computed with the M06-2X density functional^73^ and the SMD continuum solvation model^74^ to account for the effects of the solvation environment, as single-point calculations, using the def2-TZVPP basis set^70^ on all atoms. Benzene was used for SMD solvent parameters on nitroxide/benzene and nitroxide/phenylacetylene systems; whereas cyclohexane SMD solvent parameters were specified for nitroxide/benzaldehyde and nitroxide/nitrobenzene systems.

### Theory: isotropic hyperfine coupling

The ^13^C hyperfine coupling constants were computed in the gas phase at the unrestricted B3LYP level of DFT employing the EPR-III basis^75^ for C, N, H, O. We chose to use the B3LYP functional^76^ based on previously demonstrated good EPR performance for a nitroxide in aqueous solution.^1^ Hyperfine coupling constants were computed using the spin-orbit mean-field approximation SOMF(1X).^77^ For all substrate/TEMPO systems, Boltzmann weighted isotropic HFCs for nucleus *j*, *a*_iso_^*j*^, are determined from low-energy intermolecular configurations, *c*_*i*_, as computed from eqn (5). *a*_iso_^*j*,*c*_*i*_^ is the isotropic HFC for nucleus *j* in geometrical configuration *c*_*i*_, Δ*G*^*c*_*i*_^ is the relative free energy of geometrical configuration *c*_*i*_, *R* is the universal gas constant, and *T* is the temperature (taken to be 298.15 K). Symmetry equivalent atoms are averaged together.
5

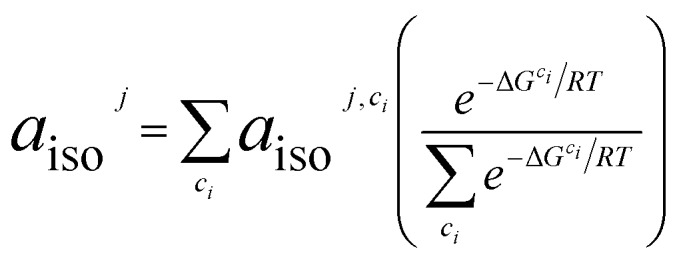




### Software

All optimization, thermochemistry, and solvation computations were accomplished using the Gaussian09 Rev D.01 suite of electronic structure programs.^78^ All EPR computations were accomplished in the gas phase using the ORCA 3.0.2 suite^79^ of electronic structure programs.

## Results and discussion

### LLIT ^1^H DNP experiments in supercritical fluid (SF) CO_2_

**Fig. 5:**
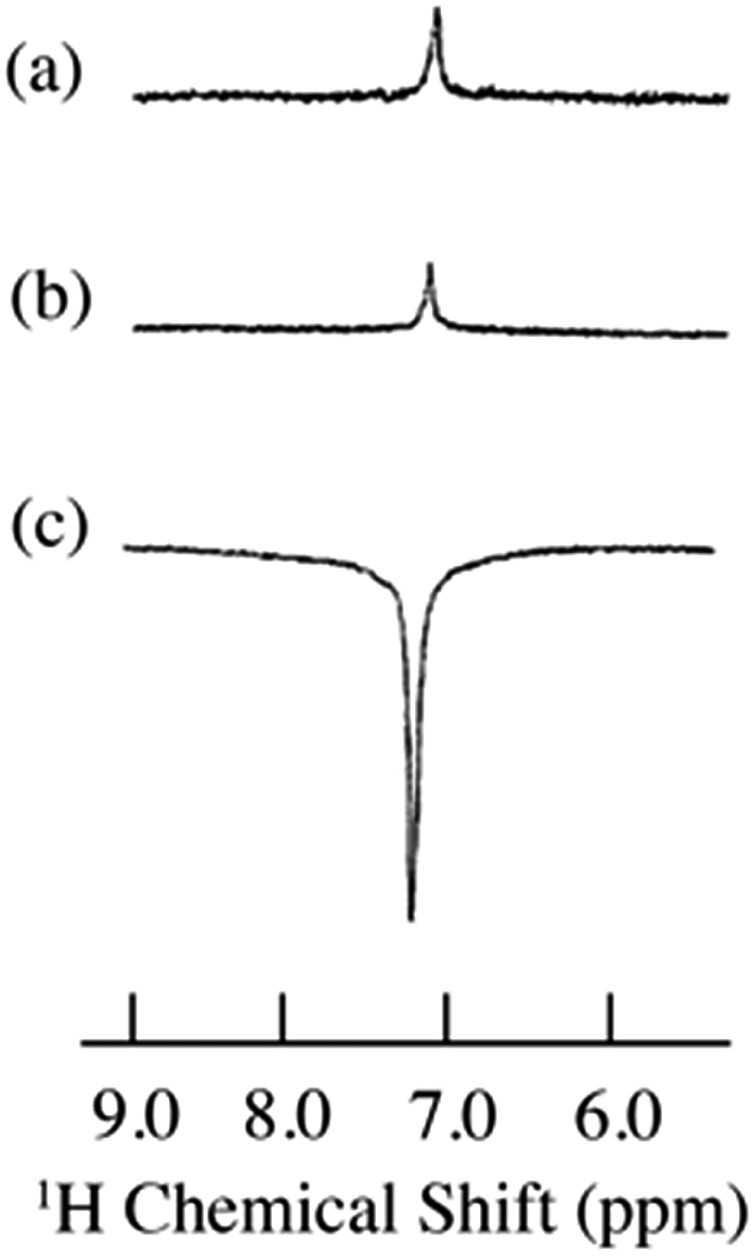
^1^H spectra of 9% benzene/0.001 M TEMPO dissolved in SF CO_2_ at 4.7 T. (a) Static ^1^H NMR (S/N = 20), (b) flow ^1^H NMR (S/N = 17) and (c) flow ^1^H DNP (S/N = 124). Total flow of SF CO_2_ was 1.1 mL min^–1^. SF CO_2_ conditions: *d* = 0.805 g mL^–1^, SFC oven temperature 40 °C, *P* = 165 atm. All spectra were collected at 32 scans.

As previously indicated, solutes dissolved in supercritical fluid (SF) CO_2_ should exhibit significant Oe DNP enhancements as a result of reduced correlation times. The static and flow ^1^H NMR, and flow ^1^H DNP spectra for 9% benzene/0.001 M TEMPO dissolved in SF CO_2_ are illustrated in Fig. 5 and the LLIT ^1^H DNP absolute enhancements of benzene dissolved in deuterated benzene and SF CO_2_ are presented in Table 1.^43^ Benzene and chloroform have been extensively used as model systems in DNP studies: both molecules present negative ^1^H DNP enhancements due to a dipolar-dominated Overhauser effect.^40^,^47^,^80^–^88^ The ^1^H DNP absolute enhancement of chloroform gives a coupling factor approaching the dipolar enhancement limit (point B in Fig. 2). An important difference of nitroxides dissolved in normal liquids *versus* supercritical fluids is the higher Heisenberg exchange rates in the latter case. Batchelor,^89^ as well as Randolph and Carlier^90^ suggest a large increase in Heisenberg electron–electron exchange rates and hence the increased line widths of nitroxides in SF's may be due to critical clustering between solute and solvent molecules. Thus, the EPR signal for TEMPO collapses to a single broad line in SF CO_2_ (see Fig. S2†). Also, the values of the saturation factor *s* are smaller in SF CO_2_ compared with normal liquids (see Table S2† and saturation curves Fig. S3 and S4†). Han *et al.*^91^ and Bennati *et al.*^6^ have presented new models and alternative methods for quantifying the Overhauser DNP saturation factor especially for the case of low radical concentrations (∼0.001 M). However, their results suggest that at nitroxide concentrations of 0.01–0.02 M the errors in determining the saturation factor are not as significant. Thus, we have presented data in liquid C_6_D_6_ determined at both 0.001 M and 0.01 M of TEMPO concentrations as illustrated in Table 1 for comparison with the SF CO_2_ data.

**Table 1:** LLIT ^1^H DNP enhancements polarized at 0.33 T for benzene/TEMPO in C_6_D_6_ and SF CO_2_

System	Absolute enhancement
9% benzene/C_6_D_6_/0.001 M TEMPO	–120 ± 18
9% benzene/SF CO_2_/0.001 M TEMPO	–341 ± 36
9% benzene/C_6_D_6_/0.01 M TEMPO	–146 ± 20
9% benzene/SF CO_2_/0.01 M TEMPO	–293 ± 44

The increased ^1^H DNP enhancement of benzene in SF CO_2_ in comparison with benzene in liquid C_6_D_6_ is attributed to increased mobility of benzene provided by the supercritical fluid. There is an approximately 2–3 fold increase in the DNP enhancement of SF CO_2_ compared with liquid C_6_D_6_, which is in agreement with the decreased factor of molecular correlation times of small molecules in SF CO_2_.^62^,^92^,^93^ Assuming a rotationally dominated mechanism, predicts correlation times of 10 and 42 ps, respectively for SF CO_2_ and liquid C_6_D_6_.^43^ Furthermore, within experimental error the absolute enhancement of 9% benzene in SF CO_2_ with 0.001 M TEMPO approaches the dipolar limiting value of –330 corresponding to a coupling factor *ρ* = 0.5, leading to nearly the highest possible dipolar-dominant enhancement in the ^1^H DNP experiment (point A in Fig. 2). This indicates that fast molecular motion of benzene in SF CO_2_ satisfies the extreme narrowing limit *ω*_S_^2^*τ*_c_^2^ ≪ 1 shown as the flat region at the low magnetic field strength in Fig. 2, for the pure dipolar relaxation models at 0.33 T. Based on discussion in the introduction for cases where the enhancement is induced by mixed scalar and dipolar relaxation, it is clear that by conducting flow transfer ^1^H DNP using SF CO_2_, both dipolar and scalar relaxation can result in substantial DNP Oe enhancements.

### SLIT ^1^H DNP experiments in supercritical fluid (SF) CO_2_

The solid–liquid intermolecular transfer (SLIT) ^1^H DNP experiment has the advantage of the radical not being present in the liquid or fluid. We have obtained SLIT ^1^H DNP results for benzene in supercritical CO_2_ with two immobilized radicals that have been previously reported.^53^Table 2 presents the SLIT ^1^H DNP absolute enhancements of benzene dissolved in deuterated benzene and SF CO_2_ using the immobilized nitroxides^43^ (I) and (II) as illustrated in Fig. 3. The absolute enhancements were obtained by estimating a transfer efficiency of ∼80% (the efficiency with which the polarized sample bolus is transferred from the low to high field strength magnet).^43^ For I and II, a 3 to 4 fold increase is observed for the ^1^H SLIT DNP enhancement for SF CO_2_, in comparison with liquid C_6_D_6_, which is due to an increase in molecular motion of the immobilized nitroxide/benzene complex in SF CO_2_. The absolute DNP enhancement from nitroxide II is slightly higher (*A* = –246) in comparison with radical I (*A* = –205) presumably due to the increased mobility (shorter correlation time *τ*_c_) of the radical attached to a longer flexible chain off the surface of the silica gel for nitroxide II.

**Table 2:** SLIT ^1^H DNP enhancements polarized at 0.33 T for benzene/nitroxide in C_6_D_6_ and SF CO_2_

System	Absolute enhancement
9% benzene/C_6_D_6_/nitroxide (I)	–67 ± 15
9% benzene/SF CO_2_/nitroxide (I)	–205 ± 24
9% benzene/C_6_D_6_/nitroxide (II)	–61 ± 17
9% benzene/SF CO_2_/nitroxide (II)	–246 ± 36

### LLIT ^13^C DNP experiments for molecular fullerene–nitroxide systems

**Fig. 6:**
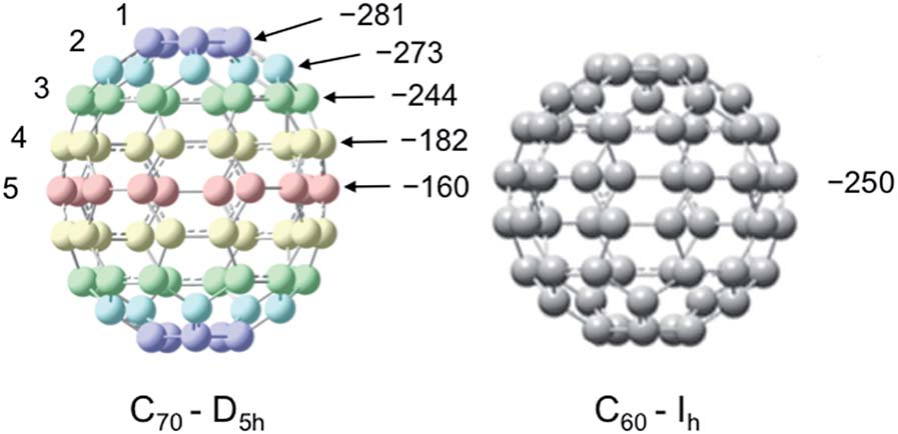
Structures of C_70_–*D*_5h_ and C_60_–*I*_h_ and LLIT ^13^C DNP enhancements polarized at 0.33 T for different carbons of C_70_/C_6_D_6_/0.1 M TEMPO system and the carbon of C_60_/C_6_D_6_/0.1 M TEMPO system. Carbon atoms of C_70_ are numbered from the apical to the equatorial positions. The relative standard deviation for *A* is about 15% (see Table S3†).

For the case of molecules containing the ^13^C nuclide, the Overhauser enhancement is usually a profile of both scalar and dipolar interactions. However, for molecules with quaternary sp^2^ hybridized carbon atoms, a dipolar enhancement usually dominates. Our laboratory previously reported LLIT and SLIT ^13^C DNP results for the fullerene C_60_/TEMPO system and observed dipolar dominated enhancements in both experiments.^65^ In contrast, the fullerene C_70_ with ellipsoidal *D*_5h_ symmetry is very different in comparison with C_60_, with 5 different non-equivalent carbon sites on the fullerene cage surface with significant differences in surface curvature as illustrated in Fig. 6. Johnson and coworkers have previously unambiguously assigned the five different carbons by 2D ^13^C NMR techniques.^94^

A surprising feature of the ^13^C DNP enhancements for the C_70_/TEMPO system is the significantly higher dipolar enhancements (*A* = –281) observed at the polar cap carbons C_1_ in comparison with the equatorial belt carbons C_5_, *A* = –160. Moreover, there is a trend of decreasing enhancement values from the polar carbons (C_1_) to the carbons at the equatorial position (C_5_). The dipolar dominated ^13^C DNP enhancement value *A* = –250 for C_60_ as well as the values for C_70_ are remarkably large when compared with small aromatic molecules (*e.g.* benzene, *A* = –220, discussed in the next section). The relatively large dipolar ^13^C DNP enhancements can be partially attributed to the high *I*_h_ and *D*_5h_ symmetry of C_60_ and C_70_ with corresponding relatively short rotational correlation times. However, a model of mixed rotational and translational diffusion cannot be excluded for the electron–nuclear interaction of both systems. It is well recognized that ^13^C paramagnetic NMR shifts are a sensitive probe for the electron–nuclear hyperfine (scalar) interaction without the time-dependence noted above for dipolar interaction. The contact shifts of both fullerenes (Table S4†) indicate a very minor scalar contribution. Moreover, the *T*_1_ relaxation times and corresponding similar *f* factors for these carbon sites exclude the influence of leakage factor *f* on the determination of absolute enhancements in the C_70_ carbon sites (see Table S3† for relaxation data and leakage factor).

**Fig. 7:**
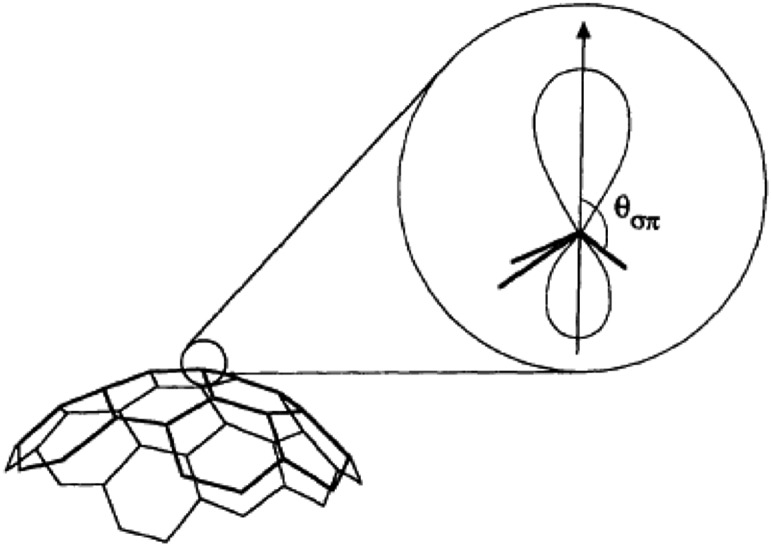
The π-orbital axis vector (POAV) is the vector of the π hybrid orbital with equal angles to the three σ bonds at a conjugated carbon atom. The average angle is denoted *θ*_σπ_. Figure reproduced from ref. 95.

**Fig. 8:**
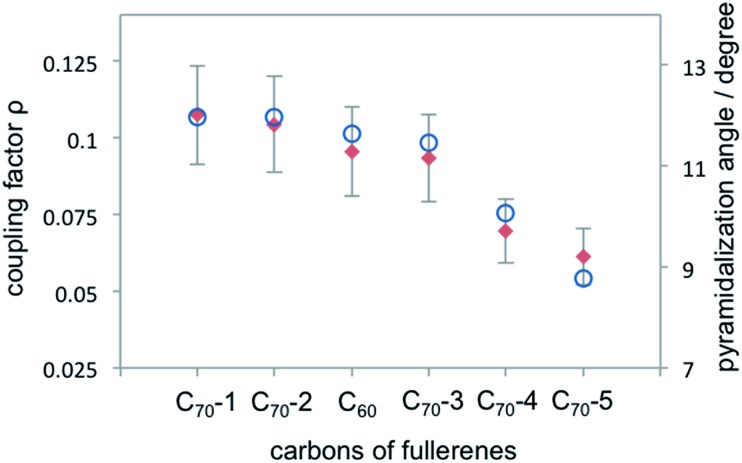
Coupling factor derived from ^13^C LLIT DNP absolute enhancements of the indicated carbons for C_70_–*D*_5h_ and C_60_ are represented by red diamond; the pyramidalization angle of fullerene carbons for C_70_ and C_60_ (ref. 95) are represented by blue circle.

One possible origin of the enhancement differences of C_70_ carbons is the variation of intermolecular distance between TEMPO and C_70_ carbon from the polar to equatorial sites. Haddon has demonstrated the importance of expressing the curvature of a surface of sp^2^ carbon atoms in terms of the π-orbital axis vector (POAV) to analyse local strain of fullerenes.^95^ The local curvature of the C_70_ carbons is assessed with the pyramidalization angle (*θ*_σπ_ – 90), where *θ*_σπ_ is the angle between the carbon σ orbital and the π orbital as shown in Fig. 7.^95^ The curvature/POAV angle is related to the interaction distance between nitroxide radicals and the carbons of C_60_ and C_70_. The C_70_ carbons with a larger pyramidalization angle are more accessible to the nitroxide radical in the weak intermolecular collisional complex, therefore resulting in a larger dipolar interaction. The correlation between the coupling factor *ρ* and the pyramidalization angle shown in Fig. 8 provides evidence for the dependence of the dipolar interaction on local curvature of the C_70_ carbons.

### LLIT ^13^C DNP experiments of other molecular substrate–nitroxide systems and density functional theory computations: background

**Fig. 9:**
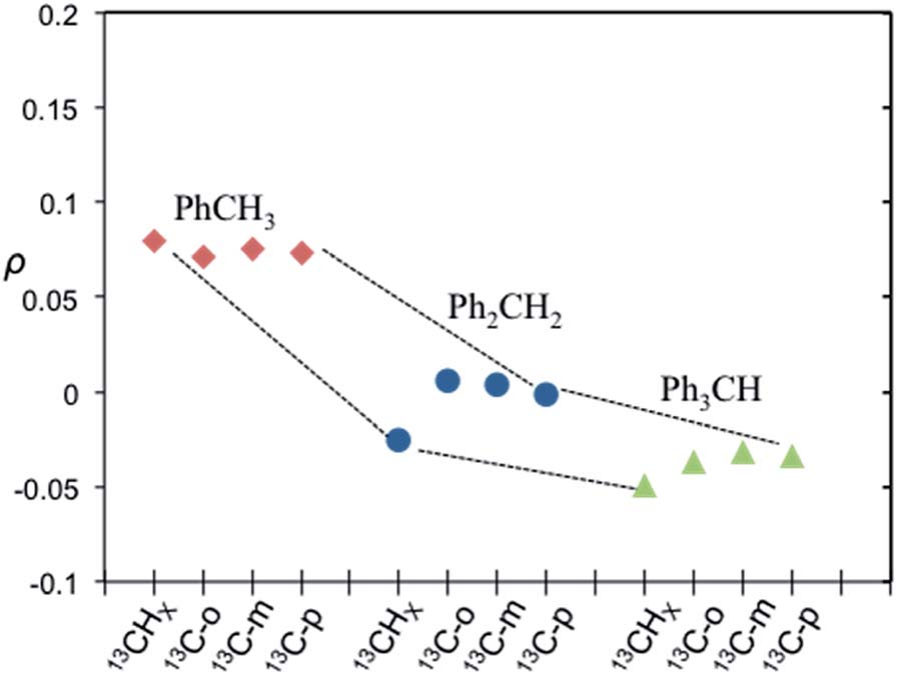
Coupling factor derived from ^13^C LLIT DNP absolute enhancements of the indicated carbon (*i.e.*^13^CH_*X*_, *X* = 1–3, ^13^C-*ortho*, ^13^C-*meta*, and ^13^C-*para*) for the series of toluene (red diamond), diphenylmethane (blue circle), and triphenylmethane (green triangle) (Ph_*Y*_CH_*X*_, *X* = 1–3, *Y* = 4–*X*).^66^ Dashed lines illustrate motif trends for different molecules. The relative standard deviation for *ρ* is about 15%.

**Fig. 10:**
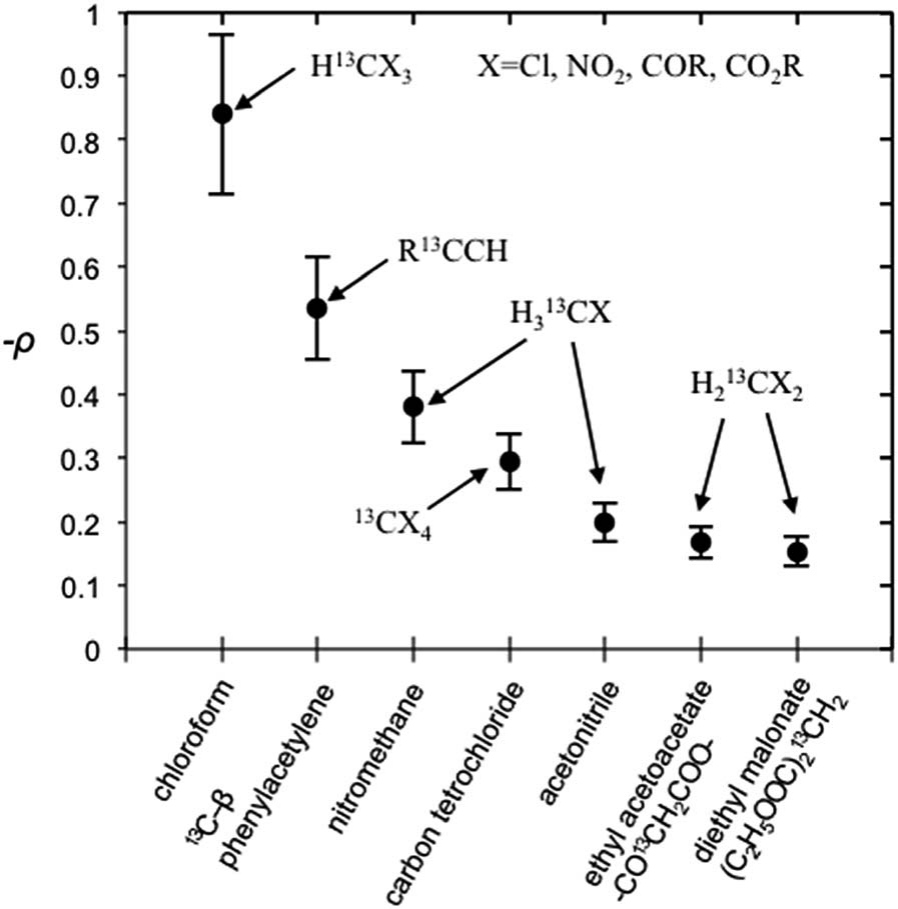
Coupling factor (negative) derived from scalar dominant ^13^C LLIT DNP absolute enhancements of small molecules. The relative standard deviation for *ρ* is about 15%.

It is well recognized that molecules with C–H groups readily form weak complexes with nitroxides provide ^13^C DNP interactions ranging from modest dipolar to strong scalar interaction(s) dependent on the hydrocarbon acidity.^44^,^46^,^50^ For the case of molecules with sp^3^ hybridized carbons (C–H) that are not bound to electronegative elements, the ^13^C DNP presents modest dipolar enhancements as illustrated for the case of cyclohexane (*A* = –270) and adamantane (*A* = –254 and –209) (Table 6). It is well recognized that there is increasing acidity for the sp^3^ hybridized carbon (–C–H_*X*_) group in progressing from toluene (p*K*_a_ = 41.2) to diphenylmethane (p*K*_a_ = 33.4), and triphenylmethane (p*K*_a_ = 31.4). This trend results in a significant change in the DNP enhancement originating from a modest dipolar (*A* = –209) to scalar interaction (*A* = +129) for ^13^CH_*X*_ (*X* = 1–3) (Fig. 9). The aromatic *ortho*, *meta* and *para* carbons reflect nearly the same dipolar to scalar trend as the sp^3^ hybridized (–C–H_*X*_) group. This trend is especially relevant in view of the change in the molecular size (and corresponding correlation time) in progressing from toluene to triphenylmethane. Clearly, spin delocalization from the sp^3^ hybridized (–C–H_*X*_) group to the aromatic pi system is an important factor dictating this trend. A second factor is the expected longer scalar correlation time for the nitroxide/triphenylmethane complex in comparison with the toluene complex, which results in a larger scalar component in the overall coupling factor. However, the sp^2^ aromatic *ipso* carbons exhibit dipolar enhancements (see Fig. S7†). The absolute enhancement trend in these cases could have a larger error because of significantly longer *T*_1_ relaxation times than the corresponding molecules with attached hydrogens (–C–H_*X*_ groups). On the other hand, for the case of sp^3^ hybridized carbons attached to electron withdrawing groups (X–C–H_*X*_), these weakly acidic hydrocarbons lead to very significant scalar enhancements as represented by chloroform (HCCl_3_) example with *A* = +2200, which is close to the scalar limit. In addition, there are numerous other examples of large scalar enhancements for electron withdrawing sp^3^ hybridized carbon (X–C–H_*X*_) groups including nitromethane, diethyl malonate, ethylacetoacetate, and acetonitrile as illustrated in Fig. 10 and Table 6. All of these examples have acidic (C–H) groups with p*K*_a_ acidities ranging from 10 to 25. Thus, sp^3^ hybridized carbon hydrocarbons exhibit a wide range of DNP enhancements ranging from those with p*K*_a_s 40–50 (*e.g.*, toluene, cyclohexane) that exhibit modest dipolar interactions, to hydrocarbons with C–H groups exhibiting p*K*_a_ acidities between 10 and 30 with large scalar enhancements (*A* = +400 to +2200). Even molecules with weakly acidic (C–H) sites exhibit significant scalar enhancements, for example, the C_1_ carbon in 1-chlorobutane (*A* = +460), when the remaining non-acidic (C–H) sites (C_2_–C_4_) exhibit dipolar enhancements (Table 6). The ability of nitroxide radicals to probe the weak acidity of C–H groups in more complex molecular systems is a unique application of solution state DNP.

### Benzene/TEMPO

**Fig. 11:**
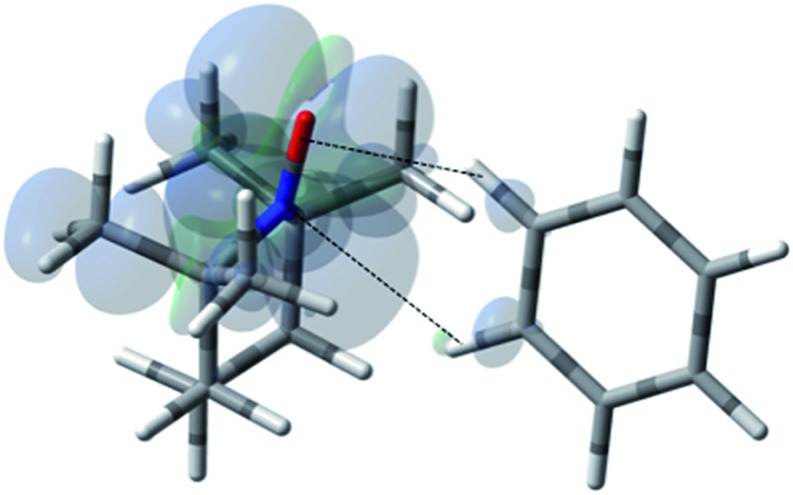
The electronic spin density distribution of the largest ^13^C–H HFC contributing orientation between benzene and TEMPO where two C–H moieties bite the TEMPO N–O group. Dashed lines are given as guides for intermolecular distances, which are 2.39 Å for O···H (of C–H moiety with closest approach to TEMPO oxygen) and 2.86 Å for N···H (of C–H with closest approach to TEMPO nitrogen).

**Fig. 12:**
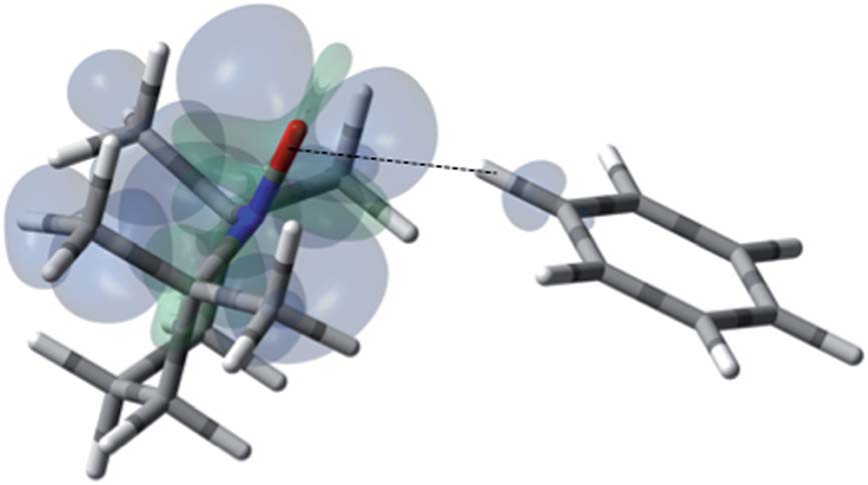
The electronic spin density distribution of the second largest ^13^C–H HFC contributing orientation between benzene and TEMPO where one C–H moiety points at N–O group. Dashed line is given as a guide for intermolecular distance, which is 2.30 Å between the oxygen atom of TEMPO and the hydrogen atom of benzene C–H moiety.

For the case of hydrocarbons with sp^2^ hybridization, benzene has been an archetypal system for many previous solution state DNP studies. In addition, earlier NMR contact shift studies have clearly established a small scalar contribution to the dipolar dominated DNP enhancement for the benzene/TEMPO system, *A* = –220.^44^,^68^,^96^,^97^ Electron withdrawing groups attached to sp^2^ hybridized quaternary carbons dramatically increase this dipolar interaction. For example, the aromatic *ipso* (quaternary) sp^2^ carbons (^13^C–X) of monosubstituted benzenes such as benzonitrile, phenylamine, benzaldehyde, anisole, and nitrobenzene exhibit dipolar enhancements with both electron donating and electron withdrawing substitutents (X) (see Fig. S5†). A similar trend is observed for sp^2^ hybridized carbonyl carbons with electronegative oxygens directly attached (Fig. S5†). The highest dipolar enhancement in this trend is for the carbonyl carbon of acetone with a dipolar enhancement of *A* = –744 representing a coupling factor of ∼55% of the dipolar limit at 0.33 T for this small molecule. For comparison, the significantly larger fullerene C_60_ molecule with sp^2^ hybridized quaternary carbons exhibits a solution state enhancement of *A* = –250 and does not have a significant scalar component *vide supra*.^65^ These results suggest that substrate molecules (100–700 Daltons) with sp^2^ hybridized carbons that do not have a significant scalar contribution exhibit dipolar enhancements with TEMPO ranging from 25 to 50% of the dipolar limit at 0.33 T. As previously noted, at the lower end of this range, the benzene/TEMPO system represents a small molecule with both dipolar and scalar ^13^C interactions. We have previously reported based on DFT calculations that the formation of weak hydrogen bonding between C–H bond of substrate molecules and nitroxides results in unpaired spin density at the substrate carbon nuclei and results in non-trivial scalar contributions to the ^13^C DNP enhancements for acetonitrile/TEMPO and acetamide/TEMPO.^49^ It is of considerable interest to establish the important orientation and interaction site(s) for the electron–nuclear interaction for the case of the benzene/TEMPO system. We have obtained the Boltzmann weighted ^13^C *a*_FC_ = 0.80 MHz *via* DFT computations using eqn (5) by considering the four lowest-energy molecular configurations (see S6–S9 and Table S5† for optimized orientations and their parameters). The two important conformations with the largest contributions to the hyperfine coupling are shown in Fig. 11 and 12. It is important to note that in both conformations the nitroxide N–O bond vector is either orthogonal to one C–H bond vector or orthogonal between (T shape) two C–H bond vectors with interaction distances of 0.230 and 0.239 nm, respectively. This orthogonal approach of the N–O and C–H bond is also consistent for the lowest energy conformations found below for other monosubstituted benzene/TEMPO complexes *vide infra*. The computational average *a*_FC_ value (*a*_FC_ = 0.80 MHz) indicates a non-trivial scalar contribution to the ^13^C DNP NMR signal of benzene. For the comparison of benzene and monosubstituted benzenes, their comparable molecular size and lack of significant conformational differences allow the assumption of relatively small changes in the correlation times.

### Nitrobenzene/TEMPO

**Fig. 13:**
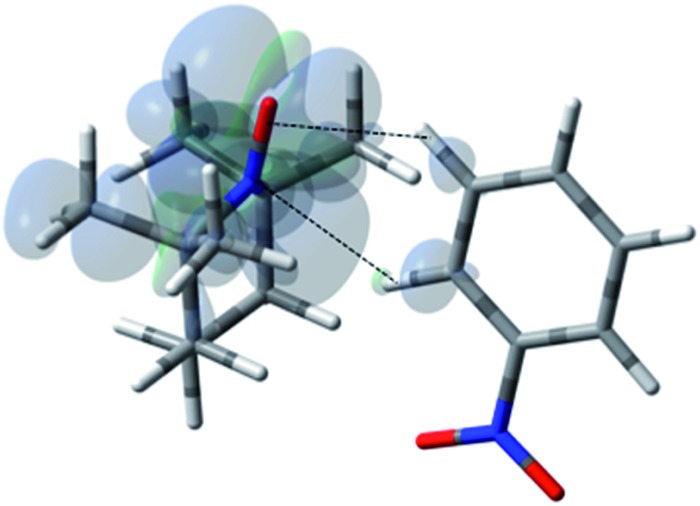
The electronic spin density distribution for the lowest energy orientation between nitrobenzene and TEMPO where *ortho* and *meta* C–H moieties complex with the TEMPO N–O group. Dashed lines are given as guides for intermolecular distances, which are 2.45 Å for O···H (of *ortho* C–H) and 2.53 Å for N···H (of *para* C–H).

**Fig. 14:**
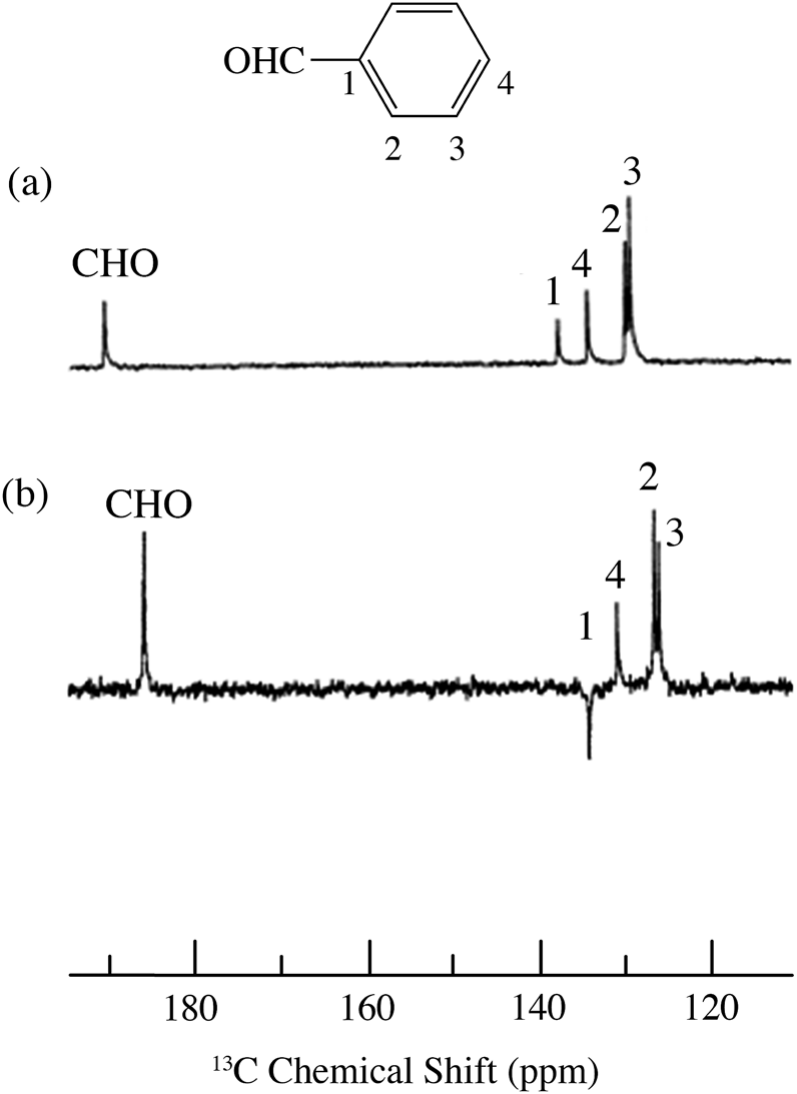
^13^C spectra of benzaldehyde/TEMPO in cyclohexane at 4.7 T (a) 0.1 M TEMPO without flow DNP, (b) 0.1 M TEMPO with flow DNP. (a) 3000 scans; (b) 735 scans.^67^

For the case of nitrobenzene/TEMPO (NMR and DNP spectra shown by Fig. 14), the introduction of the strong electron withdrawing nitro group on the aromatic ring leads to a lowest energy conformation with an orthogonal approach of the N–O and C–H bond vectors between the *ortho* and *meta* carbons (Fig. 13). The weighted Boltzmann averaging of HFC for the *ortho* carbon (*a*_FC_ = 1.424) is in strong agreement with the large scalar enhancement, *A* = +587 observed for this site (Table 3). The large ^13^C-*para* and ^13^C-*meta* contribution appears to originate from a slightly higher energy configuration (Fig. S23†) where the *para* C–H bond vector is orthogonal to the nitroxide, N–O bond vector. However, delocalization of polarized spin from the dominant *ortho* conformation to the *meta* and *para* sites can not be excluded. It is interesting to note in this regard that the strong electron withdrawing nitro group leads to scalar enhancements, whereas, electron donating groups such as the amino group of phenylamine leads to dipolar enhancements at all *ortho*, *meta*, and *para* carbon positions (Table S10†).

**Table 3:** LLIT ^13^C DNP enhancements polarized at 0.33 T for nitrobenzene/cyclohexane/0.1 M TEMPO^66^ and *a*_FC_*via* DFT modeling

System	Nucleus	Absolute enhancement	*a*_FC_ ^*a*^
Nitrobenzene	^13^C-*ipso*	–572 ± 86	0.399
	^13^C-*ortho*	+587 ± 88	1.424
	^13^C-*meta*	+198 ± 30	0.329
	^13^C-*para*	+366 ± 55	0.418

^*a*^ *a*_FC_ is the Fermi contact HFC in units of MHz computed as described.

### Benzaldehyde/TEMPO

**Fig. 15:**
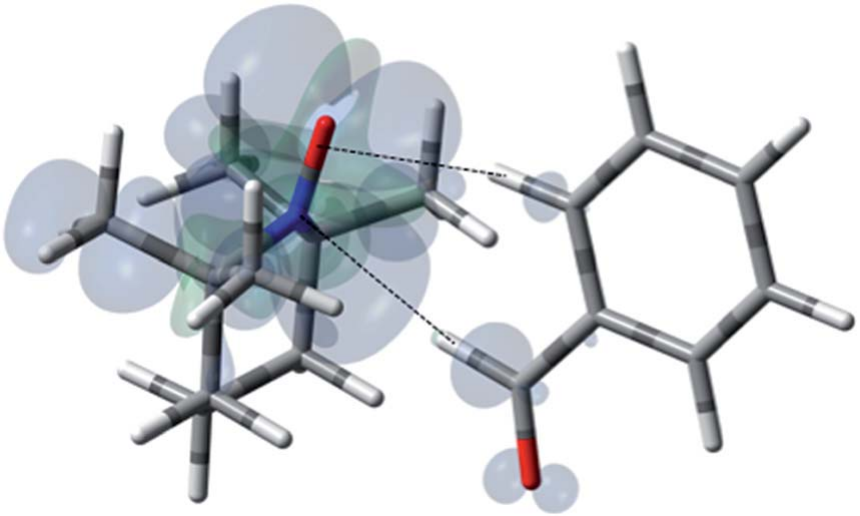
The electronic spin density distribution for the largest ^13^CHO– and ^13^C-*ortho* HFC contributing orientation between benzaldehyde and TEMPO where the aldehyde and *ortho* C–H moieties bite the N–O group. Dashed lines are given as guides for intermolecular distances, which are 2.39 Å for O···H (of *ortho* C–H) and 2.46 Å for N···H (of aldehyde C–H).

In contrast with benzene and nitrobenzene, the benzaldehyde/TEMPO system provides a system where two different weak sp^2^ hybridized C–H bonds can complex with TEMPO (Fig. 15 and Table 4). The weighted Boltzmann averaging of hyperfine couplings continues to show strong agreement with the scalar enhancements measured by DNP (see Table S7† for details). In this case the *ortho* aromatic C–H bond and the aldehyde, O=C–H bonds both contribute as a “dual complexation site” with TEMPO for the lowest energy conformation. In addition, this conformation is similar to the orthogonal bond vector conformations found for benzene and nitrobenzene. However, the more acidic aldehyde O=C–H site exhibits a larger hyperfine coupling (*a*_FC_ = 1.155) and corresponding larger scalar enhancement (*A* = +373) in comparison with the modest hyperfine coupling and DNP enhancement found for *ortho* C–H site, *a*_FC_ = 0.442 and *A* = +131, respectively. It is also interesting to note that the modest electron withdrawing aldehyde group leads to modest scalar enhancements at all *ortho*, *meta*, and *para* carbon positions (Table 4).

**Table 4:** LLIT ^13^C DNP enhancements polarized at 0.33 T for benzaldehyde/cyclohexane/0.1 M TEMPO^67^ and *a*_FC_*via* DFT modeling

System	Nucleus	Absolute enhancement	*a*_FC_ ^*a*^
Benzaldehyde	^13^CHO–	+373 ± 56	1.155
	^13^C-*ipso*	–456 ± 20	0.284
	^13^C-*ortho*	+131 ± 20	0.442
	^13^C-*meta*	+93 ± 14	0.063
	^13^C-*para*	+78 ± 12	0.099

^*a*^ *a*_FC_ is the Fermi contact HFC in units of MHz computed as described.

### Phenylacetylene/TEMPO

**Fig. 16:**
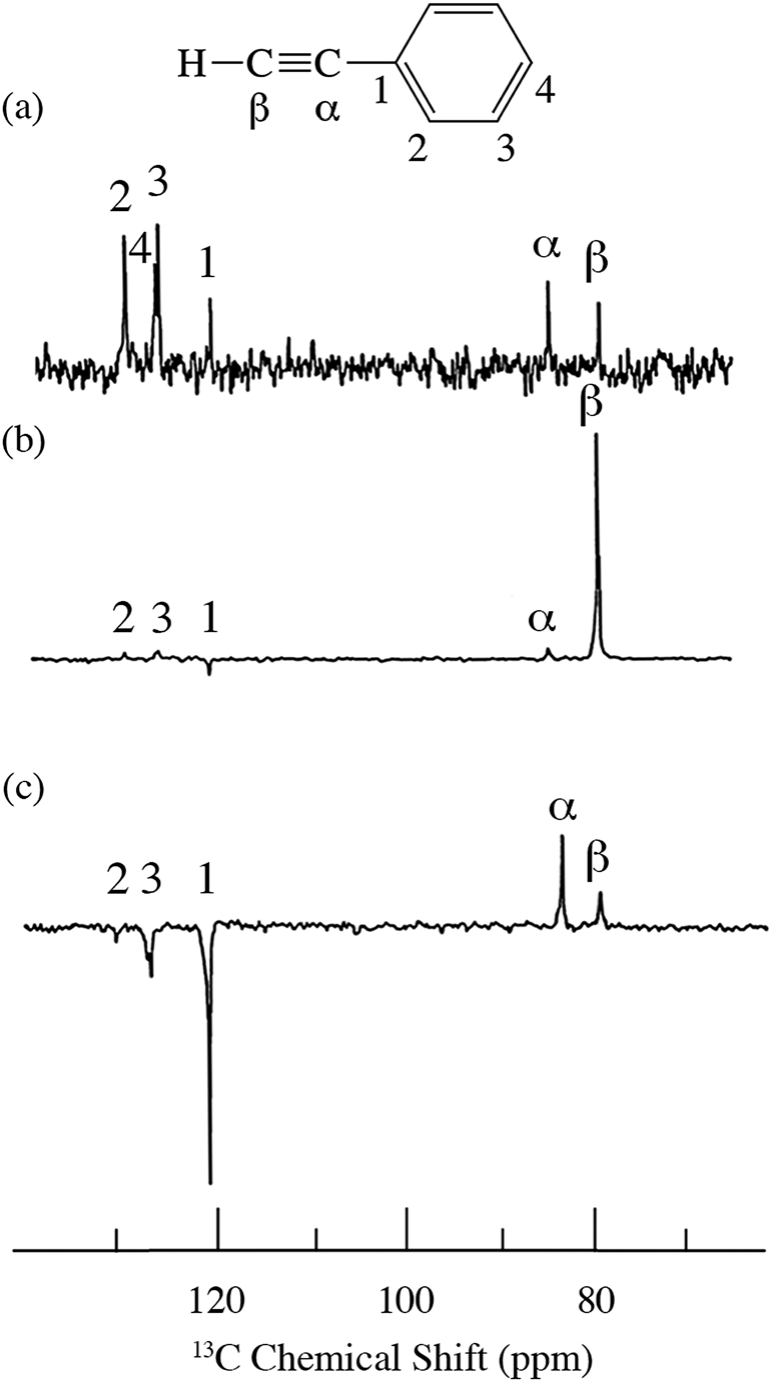
^13^C spectra of phenylacetylene/TEMPO at 4.7 T (a) 0.01 M TEMPO without flow DNP, (b) 0.01 M TEMPO with flow DNP and (c) 0.14 M TEMPO. (a) 16 scans; (b) 16 scans; (c) 64 scans.^45^

**Fig. 17:**
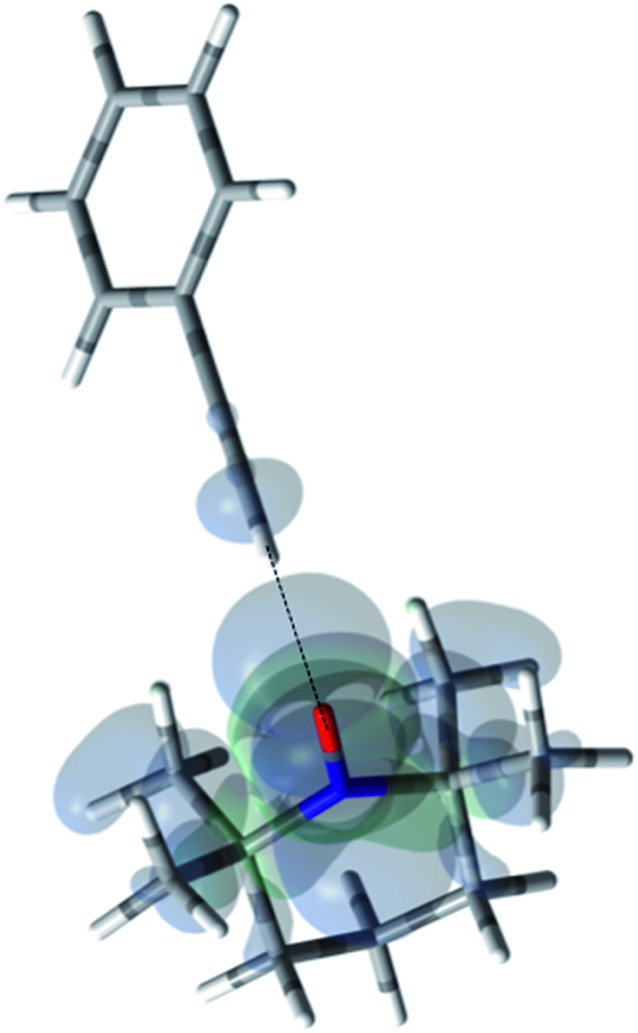
The electronic spin density distribution of the largest ^13^C-β HFC contributing orientation between phenylacetylene and TEMPO where the alkyne points at N–O group. Dashed line is given as guide for intermolecular distance, which is 2.03 Å between oxygen atom of TEMPO and the hydrogen atom of acetylene group of phenylacetylene.

For the case of sp hybridized carbons there is a paucity of data for the complexation of alkynes with nitroxides. For cyano sp hybridized carbons modest ^13^C dipolar DNP enhancements have been reported for benzonitrile and acetonitrile (Table 6 and Table S16†). However, for the acidic sp hybridized C–H carbons there is a paucity of examples reported. In one notable early NMR shift study, a transient hydrogen bonding between phenylacetylene and di-*tert*-butyl nitroxide was proposed,^68^ since the former is a good proton donor with a p*K*_a_ of 21 (Fig. 1). It should be noted that alkynes are key components in the widely employed “click” reaction extensively employed in organic, polymer, and biomedical functionalization studies.^98^ Phenylacetylene has an acetylenic proton of relatively high acidity (p*K*_a_ of 21)^99^ compared with benzene as shown in Fig. 1. The DFT and DNP results for phenylacetylene/TEMPO are shown in Fig. 17 and Table 5. As predicted, the phenylacetylene/TEMPO exhibits a large hyperfine coupling, *a*_FC_ = 3.28 and corresponding large scalar dominated enhancement (*A* = +1400) for the sp hybridized C–H β carbon (shown in Table 5). Moreover, the β carbon exhibits the largest scalar-dominant DNP enhancement observed to date for a molecule not containing halogens, corresponding to a DNP coupling factor of –0.53 (point D in Fig. 2). In analogous fashion to the previous examples above, the lowest energy conformation has the N–O bond vector orthogonal to the sp hybridized C–H bond vector (Fig. 17), but the interaction distance is considerably shorter (0.203 nm) than the previous examples, *vide supra*. It is also interesting to note that the α sp hybridized carbon exhibits a notable scalar enhancement (*A* = +290) as well, also consistent with the relatively large computationally derived hyperfine coupling (*a*_FC_ = 0.871). The *ortho*, *meta*, and *para* carbons on the benzene ring of phenylacetylene show very small dipolar-dominated (negative) enhancements that are nearly zero. Clearly there is a large difference in the spin–lattice *T*_1_ relaxation time of the scalar dominated β carbon relative to the *o*, *m*, and *p* carbons of the aromatic ring. At low radical (TEMPO) concentrations (0.01 M), the β carbon polarization is notably enhanced, but the other carbon atoms are barely observable. For the ring carbons having dipolar-dominated interactions, whereas, at higher radical concentrations (0.14 M) dipolar enhancements become observable, but the β carbon is barely observed because of the very short nuclear relaxation times at this concentration which limits the transfer efficiency from the low (0.33 T) to the high observable magnetic field (4.7 T). However, the lower radical concentration used (0.01 M) may diminish the observed DNP enhancements for the β carbon and other carbons because of a lower leakage factor and three-spin effects^100^ (Fig. 16b). These results suggest that for molecules having specific strong scalar complexation sites, it is possible to independently detect enhancements contributed by the scalar and dipolar interactions by varying the radical concentration.

**Table 5:** LLIT ^13^C DNP enhancements polarized at 0.33 T for phenylacetylene/0.001 M and 0.14 M TEMPO^45^ and *a*_FC_*via* DFT modeling

System	Nucleus	Absolute enhancement	*a*_FC_ ^*a*^
Phenylacetylene^*b*^	^13^C-β^*c*^	+1400 ± 210	3.281
	^13^C-α	+290 ± 44	0.871
	^13^C-*ipso*	–280 ± 42	0.277
	^13^C-*ortho*	–36 ± 5	0.579
	^13^C-*meta*	–32 ± 5	0.117
	^13^C-*para*	N/A^*d*^	0.181

^*a*^ *a*_FC_ is the Fermi contact HFC in units of MHz computed as described.

^*b*^Inverse gated ^1^H decoupling was employed.

^*c*^C-β results are based on measurements of 0.01 M TEMPO solution, while C-α and C-1–3 results are from 0.14 M TEMPO solution.

^*d*^C-4 signal is close to zero.

## Conclusions

In summary, we have found that for ^1^H DNP, supercritical fluids provide a convenient approach for decreasing the correlation time for solutes and provide an alternative approach for improving dipolar enhancements at high magnetic fields without the need for polarization at low temperatures (∼4 K). For the system SF CO_2_/benzene/TEMPO, the DNP enhancement approaches the dipolar limiting value of –330 (*ρ* = 0.5) at 0.33 T. For biomedical applications, there has been considerable interest in the DNP enhancement of water as reviewed by Günther.^8^ For example, Bennati and coworkers have experimentally reported an Oe dipolar DNP enhancement *A* = –170 for the water/TEMPONE-D-^15^N system at 9.7 GHz (0.33 T).^22^

**Fig. 18:**
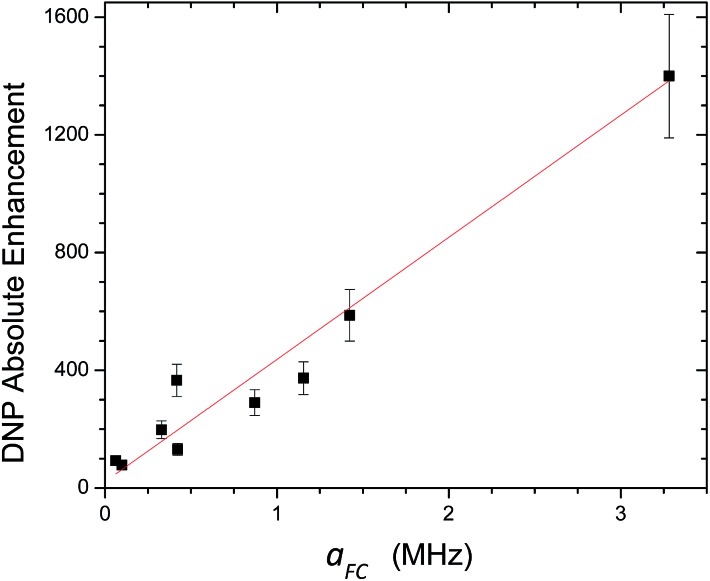
Correlation between experimentally determined ^13^C LLIT DNP scalar dominated enhancements and their calculated AFC (Boltzmann weighted DFT computed Fermi contact hyperfine coupling constant) for the phenylacetylene/TEMPO, benzaldehyde/TEMPO, and nitrobenzene/TEMPO systems. After performing a linear regression with direct weighting of the DNP enhancement error, a line of best fit (*y* = *mx* + *b*) was determined having parameters *m* = 415 ± 23, *b* = 21 ± 52, and the goodness of the fit is computed as having an adjusted *R*^2^ = 0.97.

For ^13^C DNP at 0.33 T, we have found that molecules (100–700 Daltons) dissolved in liquids containing carbon sites without a significant scalar contribution (*e.g.*, carbonyls) exhibit dipolar enhancements ranging from, *A* = –250 to –740 with TEMPO *vide supra*. It is easy to predict that ^13^C DNP for these carbon sites could also exhibit ^13^C DNP enhancements 2 to 3 times greater if conducted in SF CO_2_. For the biomedical metabolites, the enhancement in SF CO_2_ followed by rapid dissolution in water or saline solutions and transfer to high magnetic fields could be a viable approach for NMR nuclides with long *T*_1_ relaxation times for both NMR and MRI applications. Furthermore, other SF fluids, such as, supercritical water and nitrous oxide represent alternative fluids for decreasing correlation times at higher magnetic fields for DNP at high fields. For the case the C_70_/TEMPO system, we have reported significantly higher dipolar enhancements at the polar cap carbons with greater sp^2^ curvature in comparison with the equatorial belt carbons. For the case of ^13^C DNP where both dipolar and scalar interactions are important, the correlation between experimental scalar DNP enhancements (*A*) and the Boltzmann weighted hyperfine coupling constants (*a*_FC_) calculated using the DFT methods provides a predictive tool for any substrate systems exhibiting a non-trivial scalar interaction as illustrated in Fig. 18. A cautionary note of these predictions is the importance of the scalar and/or dipolar correlation time(s) that is not included in the current computational approach. For this work, we assume similar correlation times for the monosubstituted benzenes employed in this study. Finally, of critical importance, molecules with sp hybridized C–H groups, such as, the β carbon of phenylacetylene deserve further study because of the large scalar enhancements observed for this system. The short intermolecular distance between the hydrogen attached to the β C–H group and the orthogonal O–N bond of the nitroxide in TEMPO is about 2.0 Å (see Fig. 17). The shorter intermolecular distance is an indication of stronger hydrogen bonding between the acetylene group and the radical, and hence has a greater effect on the scalar interaction, compared with other sp^3^ and sp^2^ hybridized carbon systems. Furthermore, compared with sp^2^ carbons such as the aldehyde carbon of benzaldehyde, the phenylacetylene β carbon has a greater *s* character of the bonding carbon hybrid orbital caused by sp hybridization. The hyperfine coupling constant (*a*_FC_) is directly proportional to the unpaired electron spin density at the investigating carbon nucleus, hence the increased HFC value of phenylacetylene β carbon indicates the contribution of increased *s* character and corresponding greater spin density transfer from the radical to the sp carbon nucleus, |*ψ*(0)|^2^. This conclusion is consistent with the observation of experimental and computed increasing *s* character for ^13^C–^1^H coupling constants (*J*_CH_) which increases markedly as a result of Fermi contact contributions^104^ with the trend, 159 Hz for benzene,^105^ 173.7 Hz for the carbonyl group in benzaldehyde,^106^ (C_6_H_5_^13^C^1^HO) and 251 Hz for the sp β carbon in phenylacetylene^107^ (C_6_H_5_C^13^C^1^H). In conclusion, solution state DNP provides a unique approach for studying intermolecular weak bonding interactions of solutes in normal liquids and SF fluids.

**Table 6:** LLIT ^13^C DNP enhancements polarized at 0.33 T for substrate/TEMPO systems and acid dissociation constants (p*K*_a_)^101^,^102^ at 25 °C of ^13^C–H protons

System (solute/solvent/TEMPO conc.)	Nucleus	Absolute enhancement	p*K*_a_	Type of carbon hybridization
Phenylacetylene/NA/0.01 M (ref. 45)	^13^C-β	+1400 ± 210	21	sp
Phenylacetylene/NA/0.14 M (ref. 45)	^13^C-α	+290 ± 44	—	
Benzaldehyde/cyclohexane/0.1 M (ref. 67)	^13^CHO–	+373 ± 56	14.9	sp^2^
	^13^C-*ortho*	+131 ± 20	—	
Nitrobenzene/cyclohexane/0.1 M (ref. 66)	^13^C-*ortho*	+587 ± 88	—	
	^13^C-*meta*	+198 ± 30	—	
	^13^C-*para*	+366 ± 55	—	
Chloroform/0.0052 M (ref. 44)	^13^CHCl_3_	+2200 ± 330	15.5	sp^3^
Nitromethane/benzene/0.1 M (ref. 66)	^13^CH_3_NO_2_	+996 ± 149	10	
Carbon tetracholoride/1-chlorobutane/0.08M (ref. 44)	^13^CCl_4_	+770 ± 115	—	
Acetonitrile/carbon tetrachloride/0.1 M (ref. 103)	^13^CH_3_CN	+520 ± 100	25	
1-Chlorobutane/carbon tetrachloride/0.08 M (ref. 44)	Cl^13^CH_2_CH_2_CH_2_CH_3_	+460 ± 70	—	
Ethyl acetoacetate/benzene/cyclohexane/0.1 M (ref. 66)	CH_3_CO^13^CH_2_COOCH_2_CH_3_	+440 ± 66	11	
Diethyl malonate/benzene/cyclohexane/0.1 M (ref. 66)	(CH_3_CH_2_OOC)_2_^13^CH_2_	+401 ± 60	13	
Triphenylmethane/cyclohexane/0.1 M (ref. 66)	^13^CH(Ph)_3_	+129 ± 19	31.4	
Diphenylmethane/cyclohexane/0.1 M (ref. 66)	^13^CH_2_(Ph)_2_	+67 ± 10	33.4	
Benzene/0.1 M (ref. 66)	^13^C_6_H_6_	–200 ± 30	43	sp^2^
C_60_/C_6_D_6_/0.1 M (ref. 65)	^13^C_60_	–250 ± 20	—	
Benzonitrile/cyclohexane/0.1 M (ref. 67)	^13^C-*ipso*	–281 ± 42	—	
Phenylamine/cyclohexane/0.1 M (ref. 67)	^13^C-*ipso*	–318 ± 48	—	
Toluene/cyclohexane/0.1 M (ref. 66)	^13^C-*ipso*	–423 ± 63		
Benzaldehyde/cyclohexane/0.1 M (ref. 67)	^13^C-*ipso*	–456 ± 20	—	
Anisole/cyclohexane/0.1 M (ref. 67)	^13^C-*ipso*	–457 ± 69	—	
Nitrobenzene/cyclohexane/0.1 M (ref. 66)	^13^C-*ipso*	–572 ± 86	—	
Diethyl malonate/benzene/cyclohexane/0.1 M (ref. 66)	(CH_3_CH_2_OO^13^C)_2_CH_2_	–642 ± 96	—	
Ethyl acetoacetate/benzene/cyclohexane/0.1 M (ref. 66)	CH_3_COCH_2_^13^COOCH_2_CH_3_	–711 ± 107	—	
	CH_3_^13^COCH_2_COOCH_2_CH_3_	–720 ± 108	—	
Acetone/carbon tetrachloride/cyclohexane/0.1 M (ref. 66)	(CH_3_)_2_^13^CO	–744 ± 112	—	
Toluene/cyclohexane/0.1 M (ref. 66)	^13^CH_3_Ph	–209 ± 31	41.2	sp^3^
Adamantane/benzene/0.1 M (ref. 66)	–^13^CH_2_–	–209 ± 13	—	
	–^13^CH–	–254 ± 14	—	
Cyclohexane/NA/0.1 M (ref. 45)	^13^C_6_H_12_	–270 ± 40	45	
1-Chlorobutane/carbon tetrachloride/0.08 M (ref. 44)	ClCH_2_^13^CH_2_CH_2_CH_3_	–200 ± 30	—	
	ClCH_2_CH_2_^13^CH_2_CH_3_	–400 ± 60	—	
	ClCH_2_CH_2_CH_2_^13^CH_3_	–290 ± 44	—	

## Supplementary Material

Supplementary information
