# Supplementary material for: Optimization and prediction of the electron–nuclear dipolar and scalar interaction in 1H and 13C liquid state dynamic nuclear polarization
Source: Chem Sci. 2015 Jul 29;6(11):6482–95. doi: 10.1039/c5sc02499d (PMC6054052; doi:10.1039/c5sc02499d)
Supplement: Supplementary file 1 [file SC-006-C5SC02499D-s001.pdf]

Electronic Supplementary Material (ESI) for Chemical Science.
This journal is © The Royal Society of Chemistry 2015

Optimization and Prediction of the Electron-Nuclear Dipolar and Scalar Interaction in ^1H and ^{13}C Liquid State Dynamic Nuclear Polarization

X. Wang,[a] W. C. Isley III*[b], S. I. Salido[a], Z. Sun[a], L. Song[a], K. H. Tsai[a], C. J. Cramer[b], and H. C. Dorn*[a]
[a]Department of Chemistry, Virginia Tech, Blacksburg, Virginia, 24061
[b]Department of Chemistry and Minnesota Supercomputing Institute, University of Minnesota, Minneapolis, Minnesota, 55455-0431

SUPPORTING INFORMATION

CONTENTS

- Estimation of Benzene Correlation Times in SF CO_2 and Normal Solvents
- Figure S1: Apparatus for Continuous-flow HPLC-DNP-NMR Coupled with SF CO_2
- Determination of Absolute DNP Enhancement in the Flow DNP Experiments
- Ratio Method
- Table S1: Absolute ^{13}C DNP Enhancements and Relaxation Data for 0.5 M Adamantane/Benzene/0.1 M TEMPO System
- Table S2: Electron Saturation factor s of Benzene/TEMPO system in SF CO_2 and C_6D_6
- Figure S2. The EPR field plot of 9% benzene/0.001 M TEMPO in SF CO_2
- Figure S3-S4: LLIT ^1H DNP Saturation Plots of Benzene/TEMPO system in SF CO_2 and Normal Solvents
- Table S3: LLIT ^{13}C DNP Enhancements and Relaxation Data and Leakage Factor for Different Carbons of C_{70}/C_6D_6/0.1 M TEMPO and C_{60}/C_6D_6/0.1 M TEMPO Systems and Angle Strain Parameters
- Table S4: ^{13}C NMR Contact Shift of C_{70}/TEMPO/ODCB/C_6H_{12} and C_{60}/TEMPO/C_6D_6 System
- Figure S5: Coupling factor derived from dipolar dominant ^{13}C LLIT DNP absolute enhancement of the indicated ipso carbons of substituted benzenes and carbonyls of small molecules
- Table S5 and Figure S6-S9: a_{FC} via DFT Modeling and Considered Lowest-Energy Molecular Configurations of Benzene/TEMPO System
- Table S6 and Figure S10-S14: a_{FC} via DFT Modeling and Considered Lowest-Energy Molecular Configurations of Phenyl Acetylene/TEMPO System
- Table S7 and Figure S15-S20: a_{FC} via DFT Modeling and Considered Lowest-Energy Molecular Configurations of Benzaldehyde/TEMPO System
- Table S8 and Figure S21-S25: a_{FC} via DFT Modeling and Considered Lowest-Energy Molecular Configurations of Nitrobenzene/TEMPO System
- Figure S26: LLIT ^{13}C Spectra of Nitrobenzene/TEMPO System
- Table S9-S16: LLIT ^{13}C DNP Enhancements of Benzonitrile, Phenylamine, Anisole, Toluene, Diethyl malonate, Ethyl Acetoacetate, Acetone, and Acetonitrile with TEMPO
- XYZ Coordinates of Phenyl acetylene/TEMPO, Benzaldehyde/TEMPO, and Nitrobenzene/TEMPO systems.

Estimation of correlation times corresponding to benzene ^{1}H DNP coupling factors in SF CO_2 and normal sovlent by rotational diffusion modulation of dipolar interaction. For rotational diffusion modulation, the radical induced dipolar transition probabilities can be expressed as:

$$2W_1^D = 3W_0^D = W_2^D/2 = (3/10)\gamma_S^2\gamma_I^2 h^2 \tau_r \left(n_p N_e/N_p\right) d_r^{-6} J_r(\omega_i) \qquad (S1)$$

$$\tau_r = 4\pi b^3 \eta/3kT \qquad (S2)$$

$$J(\omega_i) = 1/\left(1 + \omega_i^2\tau_r^2\right) \qquad (S3)$$

τ_r is the rotational correlation time; n_p is the number of receptor nuclei bound near each electron; N_e is the unpaired electron concentration; N_p is the total nuclear concentration; d_r is the shortest distance between the nuclear and electron spins; b is the effective tumbling radius treating solute-radical complex as a sphere; $J_r(\omega_i)$ is the rotational spectral density function.

Under the assumption that rotational dipolar interaction dominates DNP enhancement, then the coupling factor can be expressed as:

$$\rho = \left(W_2^D + W_0^D\right)/\left(W_2^D + W_0^D + 2W_1^D\right) \qquad (S4)$$

By relating eq. S4 with eq. S1 and eq. S3, the coupling factor can be further simplified as eq. S5 for dipolar interaction dominant DNP enhancement by rotational diffusion modulation:

$$\rho = \frac{1}{1.4 + 0.6\left(1 + \omega_e^2\tau_r^2\right)} \qquad (S5)$$

By eq. 1 in main text, the absolute enhancement can be converted to coupling factor ρ for ^{1}H DNP of benzene in normal solvent and SF CO_2, and the corresponding rotational correlation times can be estimated using eq. S5.

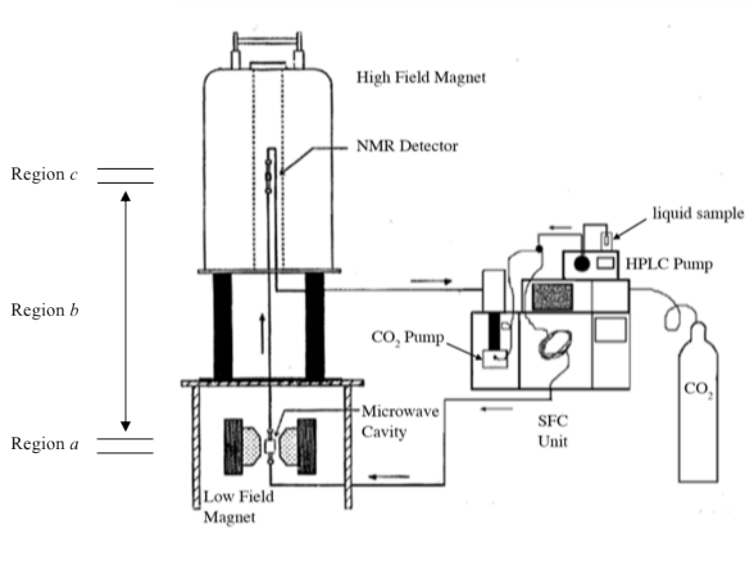

Figure S1. Schematic drawing of apparatus for continuous-flow HPLC-DNP-NMR coupled with SF CO_2 from Ref. 43.

Determination of absolute DNP enhancement in the flow DNP experiments (Model Reported in Ref. 44)

In the flow transfer DNP experiment, the nuclear spins are polarized in a low magnetic field (region *a*) and rapidly transferred via region *b*, to a high magnetic field (region *c*) where the polarization is monitored. Therefore, three separate regions (*a*, *b*, and *c* shown in Fig. S1) must be considered in calculating the enhancement factor.

The model for the DNP enhancement factor A with the consideration of spatial separation of the detection from the build-up of the signal enhancement is shown by eq. S6

$$A_{obs} = \frac{\langle M^*_Z \rangle - \langle M^{HL}_Z \rangle}{M^H_0} = \frac{[A(1 - E_{1a})]}{K}(E_{1b})(E_{1c}) \qquad (S6)$$

$\langle M^*_Z \rangle$: the enhanced magnetization detected at high magnetic field,
$\langle M^{HL}_Z \rangle$: the flow magnetization from both the low and high magnetic fields with microwave off,
M^H_0: the static magnetization detected in the high magnetic field,
A: the enhancement factor of low field DNP experiment,
K: the ratio of the two magnetic fields B^H_0/B^L_0,
$E_{1a} = \exp\left(- t_a/T_{1a}\right)$,
$E_{1b} = \exp\left(- t_b/T_{1b}\right)$,
$E_{1a} = \exp\left(- t_c/T_{1c}\right)$,
t_a, t_b, t_c: the residence times of a given bolus in regions *a*, *b*, and *c*.

In this model, the first term on the right side of eq. S6 represents the development of the enhanced magnetization in the low magnetic field (region *a*) and the second and third terms represent the transfer losses during the sample bolus is transferred to region *b* and *c*, respectively. On the left side of eq. S6, M^H_0 can be directly measured at static high field, $\langle M^*_Z \rangle$ is the DNP enhanced magnetization experimentally measured at high field. On the other hand, $\langle M^{HL}_Z \rangle$ can be measured at high field with field turned on but microwave irradiation turned off, so that it counts the transfer loss in region b and c. With M^H_0, $\langle M^*_Z \rangle$ and $\langle M^{HL}_Z \rangle$ measured experimentally, one can obtain A_{obs} values at varied flow rates.

To describe flow experiments and estimate residence times (t_a, t_b, t_c), assuming a simple plug flow pattern, where $t_a = V_a/F$, $t_b = V_b/F$, $t_c = V_c/F$, with F being the flow rate and V_a, V_a, V_a being volumes of region *a*, *b*, *c*. Then eq. S6 can be rewritten as eq. S7. By fitting a set of A_{obs} values at different flow rates, A, V_a/T_{1a}, and ($V_b/T_{1b} + V_c/T_{1c}$) can be obtained.

$$A_{obs} = \left(\frac{A}{K}\right)\left(1 - exp\left(- \frac{V_a 1}{T_{1a}F}\right)\right)exp\left(- \left(\frac{V_b}{T_{1b}} + \frac{V_c}{T_{1c}}\right)\frac{1}{F}\right) \qquad (S7)$$

When at low field, the bolus has developed full equilibrium magnetization, in other words, when T_{1a} is small, $V_a/F > 5T_{1a}$ and E_{1a} in eq. S6 is negligible, eq. S7 can then be reduced to eq. S8 and eq. S9 in logarithm form. This condition is normally met at low flow rate and when T_{1a} is small. In this case, a plot of $\ln A_{obs}$ against $1/F$ should yield a straight line, with intercept $\ln A/K$.

$$A_{obs} = \left(\frac{A}{K}\right)exp\left(- \left(\frac{V_b}{T_{1b}} + \frac{V_c}{T_{1c}}\right)\frac{1}{F}\right) \qquad (S8)$$

$$\ln A_{obs} = \ln\left(\frac{A}{K}\right) - \left(\frac{V_b}{T_{1b}} + \frac{V_c}{T_{1c}}\right)\frac{1}{F} \qquad (S9)$$

Subsequently, the absolute enhancement factor can be readily determined by correcting the leakage factor f and saturation factor s as in a static DNP experiment.

Ratio Method

In this work, the DNP data of nitrobenzene and benzaldehyde systems were collected and processed by the ratio method. This method is advantageous where sample solutions cannot be readily studied by the above method due to high demand on instrument stability and experimental time.
The procedure of the ratio method is much simpler. The absolute DNP enhancement determined by the ratio method is given by eq. S10, where A_{∞}^{ref} is the absolute DNP enhancement of the reference nucleus; $A_I(P)$ and $A_{ref}(P)$ are the observed DNP enhancements for the respective nucleus *I* and the reference nucleus measured under microwave power P; f_I and f_{ref} are the leakage factor of the nucleus I and the reference nucleus, respectively. The *s* factor is assumed to be same for all the nuclei in a given experiment. In contrast to the first method, no flow rate plot or power plot is needed for extrapolation here.

$$A_{\infty}^{I} = A_{\infty}^{ref} \cdot \frac{A_I(P)}{A_{ref}(P)} \cdot \frac{f_{ref}}{f_I} \qquad (S10)$$

Take the ^{13}C DNP experiments for adamantane/benzene/0.1 M TEMPO system for example, firstly, the observed enhancements adamantane carbons, A_{obs}^{I} at different microwave powers are calculated according to eq. S6, using M_0^H and $\langle M_Z^{HL} \rangle$, as well as $\langle M_Z^{*} \rangle$ at microwave powers of 5W, 10W, 15W and 20W. Similarly, the set of observed enhancements at four microwave power values for the reference nucleus – benzene carbons are also obtained.

Next step is to convert the observed enhancements for both adamantane and benzene carbons to the enhancement factors $A_I(P) = A_I/K$ and $A_{ref}(P) = A_{ref}/K$ at each P values using eq. S7, so that one has the enhancement factor $A(P)$ at each microwave power for both adamantane and benzene carbons.

Since the absolute ^{13}C DNP enhancement of benzene has been determined using the first method as the reference, then at every P, one can use eq. S10 to calculate the absolute enhancement of adamantane carbons, where A_{∞}^{ref} being $A_{\infty}^{benzene}$, and f_{ref} being $f_{benzene}$. Finally the adamantane absolute enhancements reported in this study are the average of values at all microwave powers. The results are shown in Table S1.

Table S1. Absolute ^{13}C DNP Enhancements and Relaxation Data for 0.5 M Adamantane/Benzene/0.1 M TEMPO System

Carbons	T_{10} / s	T_1 / s	f	$-A_{\infty}^{I}$ (P = 5 W)	$-A_{\infty}^{I}$ (P = 5 W)	$-A_{\infty}^{I}$ (P = 5 W)	$-A_{\infty}^{I}$ (P = 5 W)	$-\bar{A}_{\infty}^{I}$
$-^{13}CH_2-$	13.0	2.25	0.827	194	207	225	209	209±13
$-^{13}CH-$	20.3	2.64	0.870	236	259	269	250	254±14

Definition of transfer efficiency

According to eq. S7, one can predict a maximum A_{obs}, with the corresponding flow rate presented by eq. S11, which can be rewritten in the form of eq. S12.

$$(F_{max})^{-1} = \left(\frac{V_a}{T_{1a}}\right)^{-1} \ln\left(1 + \frac{\left(\frac{V_a}{T_{1a}}\right)}{\left(\frac{V_b}{T_{1b}} + \frac{V_c}{T_{1c}}\right)}\right)$$

$$(F_{max})^{-1} = \left(\frac{T_{1a}}{V_a}\right) \ln(1 + J)$$

where the experimental residence time variable J is defined as:

$$J = \left(\frac{V_a}{T_{1a}}\right) \Big/ \left(\frac{V_b}{T_{1b}} + \frac{V_c}{T_{1c}}\right)$$

Therefore based on eq. S7 and eq. S13, the maximum value of A_{obs} can be written as:

$$(A_{obs})_{max} = \left(\frac{A}{K}\right)\left(\frac{J}{J+1}\right) exp(-(1/J)\ln(1+J))$$

The transfer efficiency Q is then defined as $(A_{obs})_{max}$ relative to (A/K) for flow DNP experiments as a function of the experimental residence time variable J.

$$Q = \frac{K(A_{obs})_{max}}{A}$$

For most LLIT experiments presented in this work, typical values of J are in the rage of 1.5 to 5, so that a 25-40% of the enhanced magnetization is monitored in the flow DNP experiments. Whereas transfer efficiencies of 80-90% are reached for SLIT experiments corresponding to J values typically on the order of 40.

Table S2. Electron Saturation factor *s* of Benzene/TEMPO system for LLIT ^{1}H DNP Enhancements in C_6D_6 and SF CO_2

system	saturation factor	leakage factor
9% benzene/C_6D_6/0.001 M TEMPO	0.68	0.72
9% benzene/SF CO_2/0.001 M TEMPO	0.28	0.57
9% benzene/ C_6D_6/0.01 M TEMPO	0.49	0.93
9% benzene/SF CO_2/0.01 M TEMPO	0.21	0.64

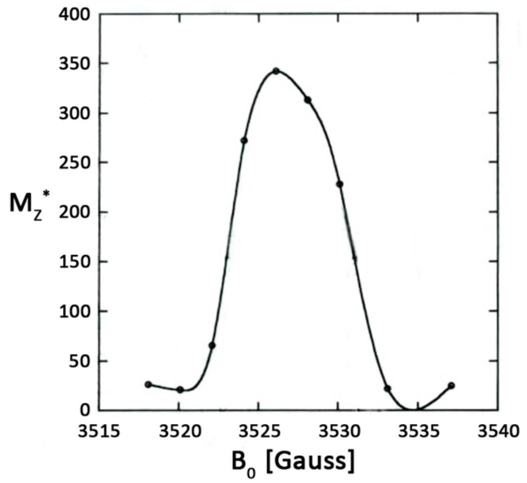

Figure S2. The EPR field plot of 9% benzene/0.001 M TEMPO in SF CO_2 collected at 9.49 GHz and 5 W of microwave power.

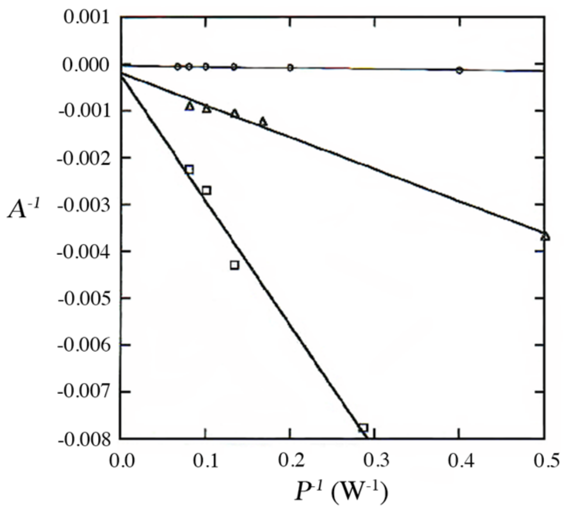

Figure S3. LLIT ^{1}H DNP saturation plot showing the inverse enhancement versus the inverse microwave power for 9% benzene in deuterated benzene (circle), neat benzene (triangle), and 9% benzene in SF CO_2 (square) in 0.001 M TEMPO. Microwave power was varied from 2 to 15 W. The value of the slope for each sample is given as follow: -2.5×10^{-4} G^2, -5.9×10^{-3} G^2, and -2.7×10^{-2} G^2 for benzene in C_6D_6, C_6H_6, and SF CO_2, respectively.

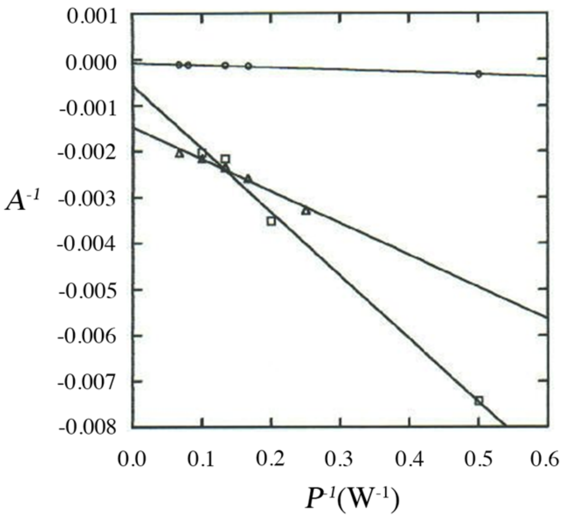

Figure S4. LLIT ^{1}H DNP saturation plot showing the inverse enhancement versus the inverse microwave power for 9% benzene in deuterated benzene (circle), neat benzene (triangle), and 9% benzene in SF CO_2 (square) in 0.01 M TEMPO. Microwave power was varied from 2 to 15 W. The value of the slope for each sample is given as follow: -5.0×10^{-4} G^2, -7.0×10^{-3} G^2, and -1.4×10^{-2} G^2 for benzene in C_6D_6, C_6H_6, and SF CO_2, respectively.

Table S3. LLIT ^{13}C DNP Enhancements and Relaxation Data and Leakage Factor for Different Carbons of C_{70}/C_6D_6/0.1 M TEMPO and C_{60}/C_6D_6/0.1 M TEMPO Systems and Angle Strain Parameters

carbon type	angle strain parameter /rad^2	T_{10}	T_1	f	absolute enhancement
C_{70}-1	0.0435	57.8	2.51	0.957	-281±42
C_{70}-2	0.0436	54.9	2.44	0.956	-273±41
C_{70}-3	0.0400	57.6	2.67	0.954	-244±37
C_{70}-4	0.0308	59.7	2.33	0.940	-182±27
C_{70}-5	0.0235	47.7	1.31	0.964	-160±24
C_{60}	0.0423	37.1	3.59	0.903	-250±38

T_1 and T_{10} are the spin-lattice relaxation times of the carbon nuclei with and without TEMPO in the sample solution, respectively.

Table S4. ^{13}C NMR Contact Shift of C_{70}/TEMPO/ODCB/C_6H_{12} and C_{60}/TEMPO/C_6D_6 System

TEMPO conc (M)	$-\Delta\delta_1$ (ppm)	$-\Delta\delta_2$ (ppm)	$-\Delta\delta_3$ (ppm)	$-\Delta\delta_4$ (ppm)	$-\Delta\delta_5$ (ppm)	$-\Delta\delta_6$ (ppm)
0.1	0.15	0.12	0.19	0.20	1.21	0.15
0.2	0.34	0.26	0.33	0.30	1.35	0.29
0.3	0.50	0.51	0.49	0.51	1.53	0.48
0.4	0.58	0.51	0.58	0.59	1.65	0.58
0.5	0.80	0.80	0.84	0.84	N/A*	0.82

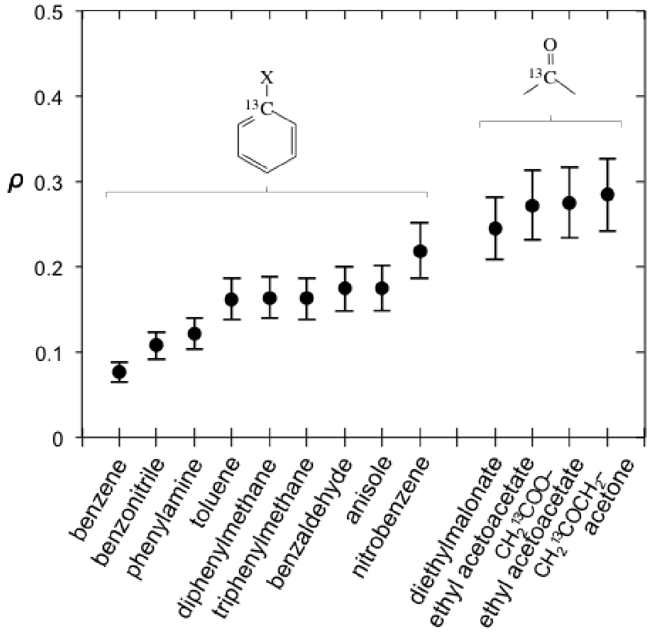

Figure S5. Coupling factor derived from dipolar dominant ^{13}C LLIT DNP absolute enhancement of the indicated *ipso* carbons of substituted benzenes and carbonyls of small molecules. The relative standard deviation for ρ is about 15%.

Table S5. LLIT ^{13}C DNP Enhancements for Benzene/C_6D_6/0.16 M TEMPO [15] and a_{FC} via DFT Modeling

Nucleus	Enhancement	a_{FC} (MHz)				
		Average	**1**	**2**	**3**	**4**
^{13}C	−220±33	0.802	0.025	0.160	0.731	0.021
			0.263	1.896	0.107	0.129
			1.046	0.163	-0.045	-0.127
			0.609	0.146	0.057	0.306
			0.120	0.029	0.862	1.658
			0.002	0.145	3.854	0.171
ΔG (kcal/mol)			0.00	0.09	0.17	3.83

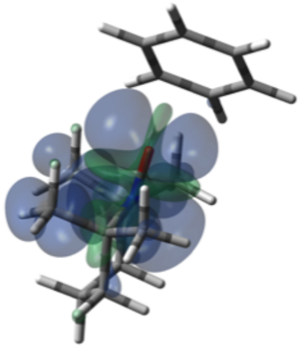

Figure S6. Interacting orientation 1 of benzene/TEMPO

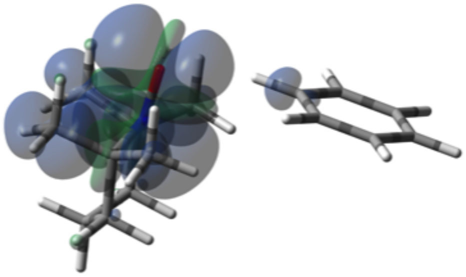

Figure S7. Interacting orientation 2 of benzene/TEMPO (Figure 12 in main text)

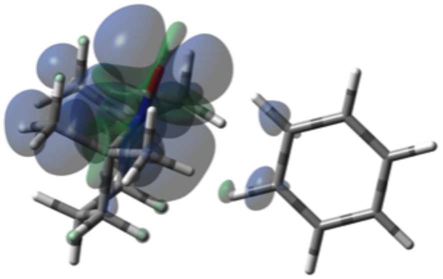

Figure S8. Interacting orientation 3 of benzene/TEMPO (Figure 11 in main text)

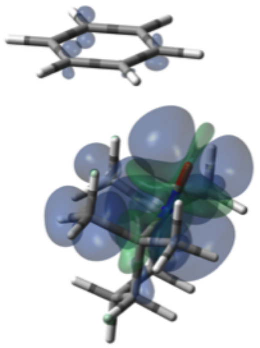

Figure S9. Interacting orientation 4 of benzene/TEMPO

Table S6. LLIT ^{13}C DNP Enhancements for Phenylacetylene/0.001 M TEMPO and a_{FC} via DFT Modeling

Nucleus	Enhancement	a_{FC} (MHz)						
		Average	**5**	**6**	**7**	**8**	**9**	**10**
^{13}C-β	+1400±210	3.281	0.256	15.249	-0.001	-0.031	0.066	0.043
^{13}C-α	+290±44	0.871	0.727	2.722	0.028	0.063	-0.105	-0.026
^{13}C-Ipso	−280±42	0.277	0.540	0.227	-0.004	-0.162	0.195	0.200
^{13}C-Ortho	−36±5	0.579	2.493	-0.082	0.124	0.173	0.313	1.526
			-0.019	-0.114	0.039	0.259	-0.100	0.040
^{13}C-Meta	−32±5	0.117	-0.104	0.021	0.554	-0.111	0.240	0.484
			0.028	0.010	0.299	0.836	0.172	0.099
^{13}C-Para	N/A	0.181	0.005	-0.023	1.245	-0.061	0.288	-0.058
ΔG (kcal/mol)			0.00	0.41	0.65	0.77	1.04	1.25

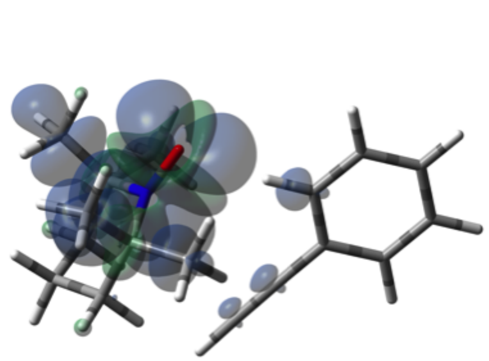

Figure S10. Interacting orientation 5 of phenylacetylene/TEMPO

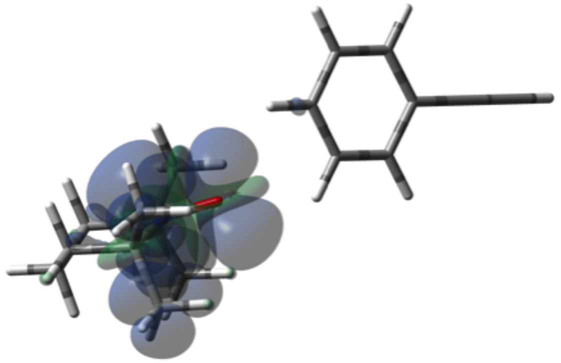

Figure S11. Interacting orientation 7 of benzene/TEMPO

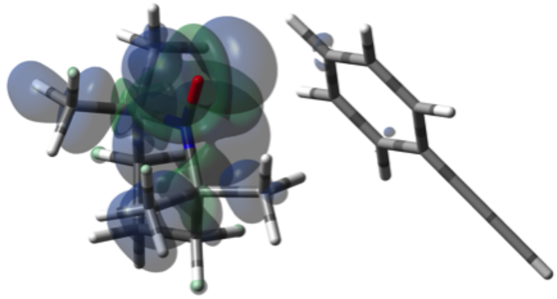

Figure S12. Interacting orientation 8 of phenylacetylene/TEMPO

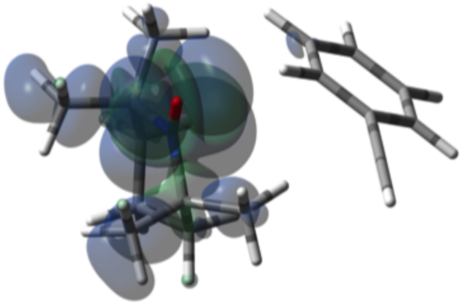

Figure S13. Interacting orientation 9 of benzene/TEMPO

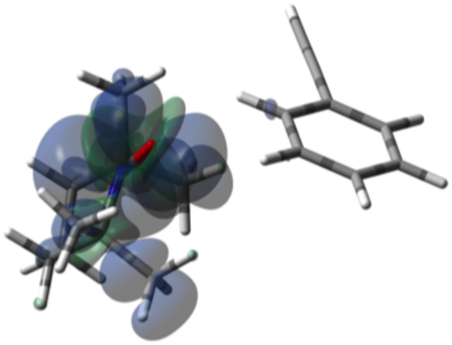

Figure S14. Interacting orientation 10 of benzaldehyde/ TEMPO

Table S7. LLIT ^{13}C DNP Enhancements for Benzaldehyde/Cyclohexane/0.1 M TEMPO and a_{FC} via DFT Modeling

Nucleus	Enhancement	a_{FC} (MHz)							
		Average	**11**	**12**	**13**	**14**	**15**	**16**	**17**
^{13}CHO-	+373±56	1.155	-0.356	1.294	-0.178	7.506	0.027	0.003	0.016
^{13}C-Ipso	−456±20	0.284	0.731	-0.003	-0.083	0.277	0.149	0.147	-0.035
^{13}C-Ortho	+131±20	0.442	1.532	0.173	0.178	1.580	0.158	0.083	0.048
			-0.219	0.229	0.204	-0.158	-0.038	0.785	0.240
^{13}C-Meta	+93±14	0.063	0.206	-0.237	0.010	-0.140	2.632	0.079	-0.135
			0.231	-0.237	0.050	0.156	0.183	0.567	0.405
^{13}C-Para	+78±12	0.099	-0.154	0.267	-0.026	0.120	0.177	0.118	2.320
ΔG (kcal/mol)			0.00	0.10	0.50	0.62	1.48	1.57	1.60

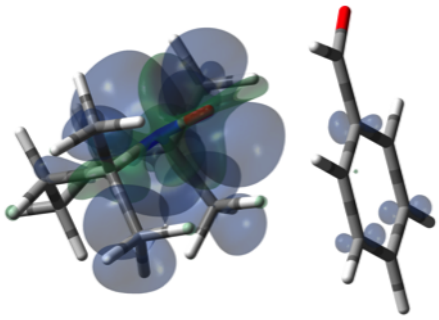

Figure S15. Interacting orientation 11 of benzldehyde/ TEMPO

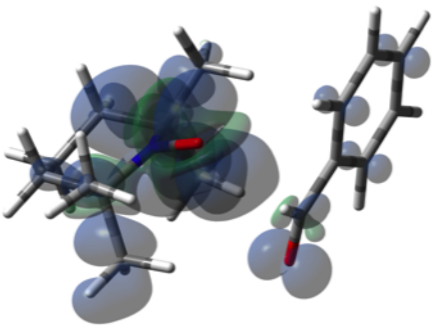

Figure S16. Interacting orientation 12 of benzaldehyde/ TEMPO

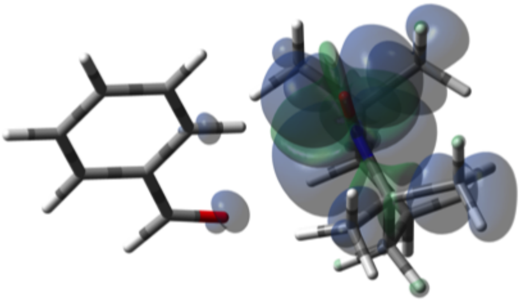

Figure S17. Interacting orientation 13 of benzldehyde/ TEMPO

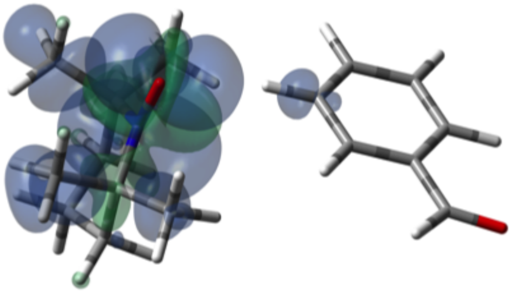

Figure S18. Interacting orientation 15 of benzaldehyde/ TEMPO

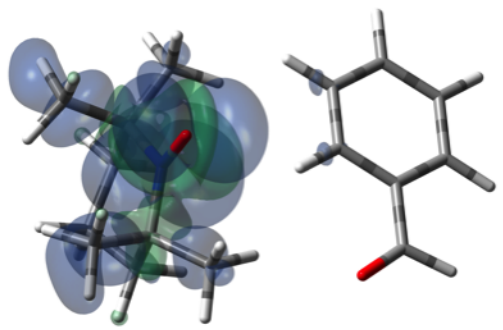

Figure S19. Interacting orientation 16 of benzldehyde/ TEMPO

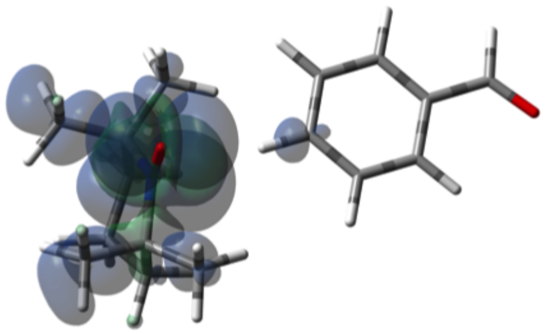

Figure S20. Interacting orientation 17 of benzaldehyde/ TEMPO

Table S8. LLIT ^{13}C DNP Enhancements for Nitrobenzene/Cyclohexane/0.1 M TEMPO and a_{FC} via DFT Modeling

Nucleus	Enhancement	a_{FC} (MHz)						
		Average	**18**	**19**	**20**	**21**	**22**	**23**
^{13}C-Ipso	−572±86	0.399	0.792	0.176	0.601	0.040	0.138	0.036
^{13}C-Ortho	+587±88	1.424	6.549	1.816	0.801	0.218	0.901	0.029
			0.112	0.024	0.476	0.035	0.048	0.007
^{13}C-Meta	+198±30	0.329	1.638	-0.070	-0.436	1.174	0.743	0.958
			-0.077	0.030	-0.403	0.340	0.107	0.027
^{13}C-Para	+366±55	0.418	0.558	-0.022	0.490	1.253	0.166	0.006
ΔG (kcal/mol)			0.00	0.05	0.42	0.49	0.85	1.01

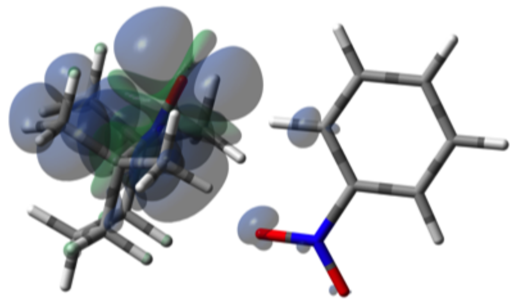

Figure S21. Interacting orientation 19 of nitrobenzene/ TEMPO

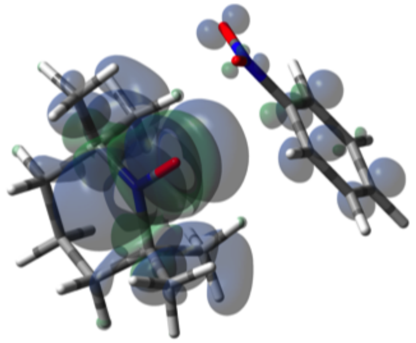

Figure S22. Interacting orientation 20 of nitrobenzene/ TEMPO

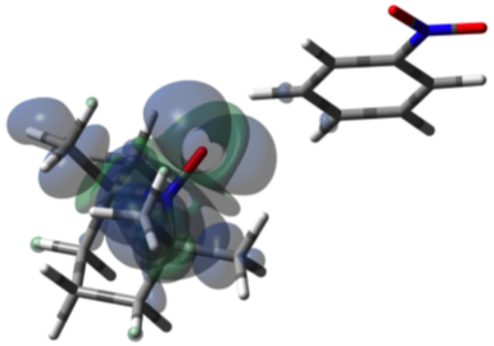

Figure S23. Interacting orientation 21 of nitrobenzene/ TEMPO

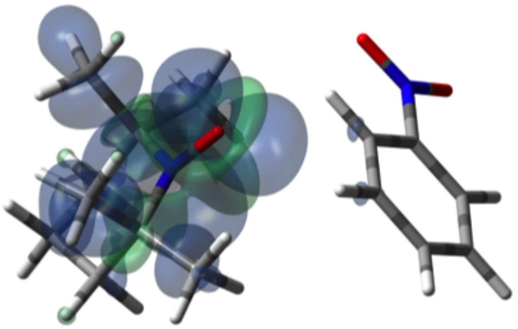

Figure S24. Interacting orientation 22 of nitrobenzene/ TEMPO

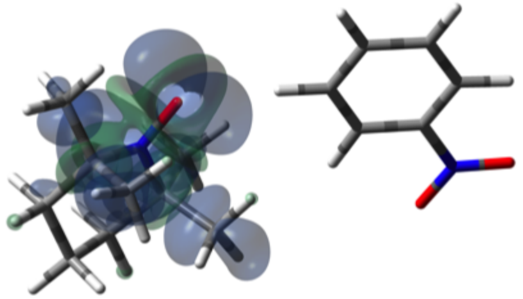

Figure S25. Interacting orientation 23 of nitrobenzene/ TEMPO

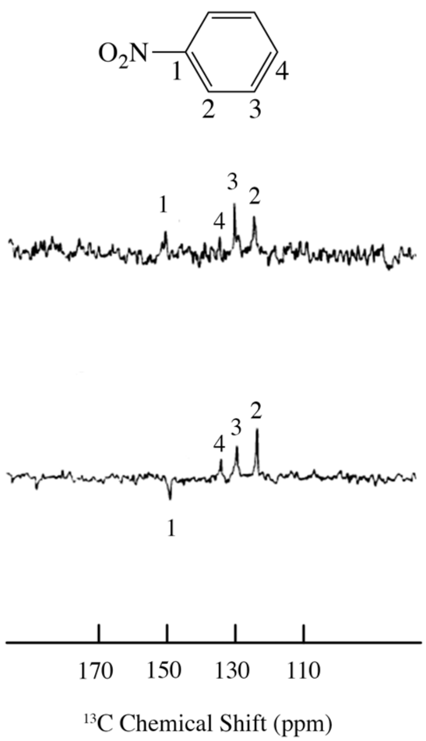

Figure S26. ^{13}C spectra of nitrobenzene/TEMPO in cyclohexane at 4.7 T (a) 0.1 M TEMPO without flow DNP, (b) 0.1 M TEMPO with flow DNP (a) 100 scans; (b) 100 scans.

Table S9. LLIT ^{13}C DNP Enhancements for Benzonitrile/Cyclohexane/0.1 M TEMPO

system	nucleus	absolute enhancement
benzonitrile	^{13}CN-	−204±31
	^{13}C–ipso	−281±42
	^{13}C–ortho	+171±26
	^{13}C–meta	+98±15
	^{13}C–para	+22±2

Table S10. LLIT ^{13}C DNP Enhancements for Phenylamine/Cyclohexane/0.1 M TEMPO

system	nucleus	absolute enhancement
phenylamine	^{13}C–ipso	−318±48
	^{13}C–ortho	−22±2
	^{13}C–meta	−131±20
	^{13}C–para	−145±22

Table S11. LLIT ^{13}C DNP Enhancements for Anisole/Cyclohexane/0.1 M TEMPO

system	nucleus	absolute enhancement
anisole	$O^{13}CH_3$-	+266±31
	^{13}C–ipso	−457±69
	^{13}C–ortho	−42±6
	^{13}C–meta	−61±9
	^{13}C–para	−64±10

Table S12. LLIT ^{13}C DNP Enhancements for Toluene/Cyclohexane/0.1 M TEMPO

system	nucleus	absolute enhancement
toluene	$^{13}CH_3$-	−187±28
	^{13}C–ipso	−562±84
	^{13}C–ortho	−179±27
	^{13}C–meta	−153±23
	^{13}C–para	−207±31

Table S13. LLIT ^{13}C DNP Enhancements for Diethyl malonate/Benzene/Cyclohexane/0.1 M TEMPO

system	nucleus	absolute enhancement
diethyl malonate	^{13}C-1	−642±96
	^{13}C–2	+401±60
	^{13}C-1′	+86±13
	^{13}C–2′	−97±14

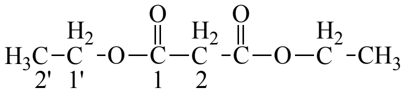

Table S14. LLIT ^{13}C DNP Enhancements for Ethyl acetoacetate/Benzene/Cyclohexane/0.1 M TEMPO

system	nucleus	absolute enhancement
ethyl acetoacetate	^{13}C-1	−711±107
	^{13}C–2	+440±66
	^{13}C–3	−720±108
	^{13}C–4	+73±11
	^{13}C-1′	+250±38
	^{13}C–2′	−117±18

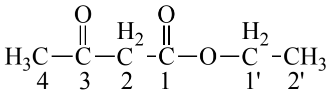

Table S15. LLIT ^{13}C DNP Enhancements for Acetone/Carbon tetrachloride/Cyclohexane/0.1 M TEMPO

system	nucleus	absolute enhancement
acetone	$-^{13}CO-$	−84±13
	$^{13}CH_3-$	−744±112

Table S16. LLIT ^{13}C DNP Enhancements for Acetonitrile/Carbon tetrachloride/0.1 M TEMPO

system	nucleus	absolute enhancement
acetonitrile	$^{13}CH_3-$	+520±100
	$^{13}CN-$	−620±120

XYZ Coordinates of Phenyl acetylene/TEMPO, Benzaldehyde/TEMPO, and Nitrobenzene/TEMPO systems.

Orientation #**5**

phenylacetylene/bite-orthometa/phenylacetylene-tempo-bite-orthometa.freq.out

C -4.754403 -1.200578 0.000110
C -3.413655 -1.585041 -0.000026
C -2.402665 -0.628710 -0.000133
C -2.731181 0.740333 -0.000104
C -4.087367 1.120713 0.000029
C -5.087346 0.154859 0.000137
H -5.542373 -1.957332 0.000190
H -3.145891 -2.643984 -0.000050
H -1.355521 -0.942381 -0.000251
H -4.342310 2.182544 0.000044
H -6.135644 0.462184 0.000234
C -1.713248 1.738418 -0.000226
C 1.899014 -0.244690 -1.324793
C 2.420871 1.190285 -1.234489
C 3.259585 1.451286 -0.000084
C 2.420815 1.190507 1.234329
C 1.898959 -0.244455 1.324874
H 2.979130 1.409000 -2.158857
H 4.164084 0.818819 -0.000002
H 3.627701 2.488500 -0.000166
H 2.979033 1.409400 2.158680
H 1.555605 1.876041 1.230067
H 1.555665 1.875826 -1.230397
C 3.005250 -1.220397 -1.729933
H 2.639035 -2.252733 -1.647627
H 3.308030 -1.044305 -2.772051
H 3.902816 -1.125363 -1.103288
C 0.757793 -0.315454 -2.330708
H 1.108279 0.026389 -3.314634
H 0.380340 -1.340065 -2.430214
H -0.076678 0.330869 -2.022102
C 3.005179 -1.220079 1.730249
H 3.307835 -1.043838 2.772377
H 2.638989 -2.252433 1.648049
H 3.902817 -1.125110 1.103695
C 0.757692 -0.315036 2.330765
H 0.380315 -1.339646 2.430559
H 1.108095 0.027142 3.314604
H -0.076823 0.331118 2.021917
N 1.350228 -0.678012 0.000075
O 0.699045 -1.769467 0.000157
C -0.864323 2.610853 -0.000346
H -0.132316 3.394304 -0.000461

Orientation #**6**

phenylacetylene/rcontact/phenylacetylene-tempo-rcontact.freq.out

C 6.501361 -0.189869 0.653996
C 5.551680 -1.138868 1.034059
C 4.238857 -1.037618 0.585975
C 3.851179 0.022246 -0.255957
C 4.818504 0.973618 -0.632782
C 6.128999 0.864823 -0.180133

H 7.531594 -0.272641 1.007442
H 5.837208 -1.967442 1.686379
H 3.494085 -1.779490 0.881349
H 4.524401 1.797512 -1.286106
H 6.867960 1.610756 -0.481767
C 2.508339 0.132447 -0.718954
C -2.673368 1.306758 0.315628
C -3.914886 1.028884 1.166052
C -3.978430 -0.389514 1.694506
C -3.992083 -1.356646 0.528597
C -2.757211 -1.246802 -0.368903
H -3.941506 1.767053 1.983392
H -3.125404 -0.595773 2.363226
H -4.877368 -0.526107 2.314029
H -4.075217 -2.400934 0.869499
H -4.890694 -1.164050 -0.082985
H -4.812829 1.221045 0.553330
C -1.419026 1.433346 1.182009
H -0.519943 1.490736 0.552922
H -1.468528 2.351437 1.784889
H -1.292792 0.591071 1.875339
C -2.868664 2.587538 -0.483693
H -3.086743 3.419500 0.200184
H -1.972215 2.836485 -1.062679
H -3.708638 2.490921 -1.185211
C -1.525714 -1.864987 0.295558
H -1.640109 -2.956635 0.359788
H -0.623392 -1.653716 -0.294813
H -1.358462 -1.490722 1.314394
C -3.018741 -1.941723 -1.697889
H -2.131058 -1.920438 -2.340083
H -3.297057 -2.989443 -1.518278
H -3.841062 -1.456788 -2.241604
N -2.478361 0.194335 -0.668849
O -1.684815 0.426271 -1.632976
C 1.364837 0.233744 -1.125024
H 0.341019 0.322889 -1.465155

Orientation #**7**
phenylacetylene/para/phenylacetylene-tempo-paraCH.freq.out
C 2.220912 0.989135 -0.748176
C 2.340055 0.395267 0.509680
C 3.571190 -0.080239 0.947711
C 4.710854 0.030761 0.127754
C 4.579916 0.631650 -1.138377
C 3.344001 1.105287 -1.567643
H 1.242855 1.351981 -1.072368
H 1.452438 0.310839 1.140896
H 3.669885 -0.544222 1.931471
H 5.460980 0.720407 -1.777422
H 3.257969 1.570092 -2.552805
C 5.974178 -0.456384 0.571748
C -3.214391 0.981036 0.355988

C -4.411143 0.346786 -0.355389
C -4.404190 -1.167580 -0.314433
C -3.143330 -1.678378 -0.981131
C -1.858068 -1.188101 -0.311155
H -5.331291 0.760156 0.087688
H -4.471121 -1.531056 0.725298
H -5.294137 -1.567293 -0.823096
H -3.116451 -2.779390 -1.007873
H -3.139337 -1.349152 -2.034793
H -4.408608 0.673585 -1.409768
C -3.354885 0.897599 1.877108
H -2.418719 1.216020 2.355674
H -4.159676 1.562035 2.222868
H -3.591004 -0.116164 2.227785
C -3.076902 2.439004 -0.060292
H -4.006433 2.979281 0.166854
H -2.249529 2.925990 0.468126
H -2.886671 2.524033 -1.139099
C -1.605149 -1.911675 1.012730
H -1.345018 -2.963563 0.826989
H -0.765019 -1.442701 1.543162
H -2.478610 -1.899983 1.678501
C -0.673046 -1.408790 -1.241242
H 0.274236 -1.139491 -0.759130
H -0.624600 -2.466915 -1.533888
H -0.770759 -0.805780 -2.154966
N -1.951890 0.283953 -0.049875
O -0.869247 0.867994 0.267017
C 7.052239 -0.872379 0.951300
H 8.002375 -1.238332 1.285808

Orientation #**8**
phenylacetylene/meta/phenylacetylene-tempo-metaCH.freq.out
C -1.436350 -1.947392 0.216167
C -2.323578 -1.627433 1.243581
C -3.354812 -0.720213 1.026074
C -3.513392 -0.109850 -0.233130
C -2.613587 -0.439876 -1.264430
C -1.587422 -1.351828 -1.036579
H -0.614891 -2.641042 0.404448
H -2.200072 -2.081863 2.229008
H -4.048439 -0.465407 1.829992
H -2.733380 0.028386 -2.243831
H -0.899312 -1.599742 -1.849464
C -4.561612 0.828308 -0.458954
C 2.549249 -0.714920 -0.444263
C 2.473098 0.097829 -1.738152
C 2.411277 1.594084 -1.507717
C 1.192036 1.922788 -0.670223
C 1.186895 1.234886 0.695962
H 3.332018 -0.179912 -2.370218
H 3.332049 1.950834 -1.014992
H 2.369042 2.126147 -2.469960

H 1.093046 3.008174 -0.508997
H 0.285816 1.612444 -1.219813
H 1.571871 -0.212682 -2.295613
C 3.933420 -0.620283 0.201826
H 3.907053 -1.067167 1.205192
H 4.672453 -1.170327 -0.398385
H 4.291653 0.413178 0.296318
C 2.236435 -2.176411 -0.732262
H 2.948107 -2.569962 -1.471244
H 2.304443 -2.783293 0.178173
H 1.224131 -2.290303 -1.143435
C 2.197425 1.868304 1.654871
H 1.859370 2.870831 1.953925
H 2.285860 1.253802 2.561402
H 3.198014 1.976994 1.215997
C -0.203170 1.318937 1.308319
H -0.233543 0.836860 2.292486
H -0.491999 2.373044 1.422903
H -0.948188 0.829727 0.666809
N 1.520966 -0.218181 0.527765
O 1.289326 -0.967742 1.525868
C -5.456075 1.629167 -0.652619
H -6.245489 2.333893 -0.822807

Orientation #**9**
phenylacetylene/ring/phenylacetylene-tempo-ring.freq.out
C 2.000319 -2.301201 0.601917
C 1.763127 -1.704511 -0.635911
C 2.462061 -0.561882 -1.008940
C 3.417553 0.001213 -0.143933
C 3.652012 -0.609592 1.103323
C 2.946082 -1.750208 1.469653
H 1.446941 -3.197466 0.894178
H 1.011660 -2.111552 -1.315049
H 2.250605 -0.079977 -1.964971
H 4.390429 -0.174041 1.779648
H 3.134017 -2.213992 2.440780
C 4.116326 1.187219 -0.511956
C -2.524447 -0.779208 -0.498197
C -3.109080 -0.904155 0.909652
C -3.252639 0.425017 1.621833
C -1.887356 1.069832 1.744816
C -1.210719 1.332115 0.397791
H -4.073321 -1.432239 0.835046
H -3.951221 1.085601 1.080417
H -3.697699 0.279039 2.617506
H -1.936987 2.024261 2.292891
H -1.235300 0.409365 2.342668
H -2.449638 -1.557133 1.507803
C -3.546437 -0.211143 -1.484526
H -3.056013 0.006896 -2.443076
H -4.349691 -0.939524 -1.666760
H -4.015184 0.713845 -1.122865

C -2.058555 -2.144813 -0.983195
H -2.900930 -2.850566 -0.976035
H -1.658115 -2.087799 -2.002438
H -1.272822 -2.544964 -0.327087
C -1.843819 2.520415 -0.328082
H -1.604195 3.457688 0.194282
H -1.445424 2.586334 -1.349725
H -2.938282 2.448136 -0.391112
C 0.271983 1.599541 0.612799
H 0.779398 1.830787 -0.331820
H 0.399709 2.452664 1.294103
H 0.767898 0.726947 1.061558
N -1.325149 0.119325 -0.476729
O -0.618771 0.124390 -1.532053
C 4.704479 2.204405 -0.825828
H 5.225045 3.099032 -1.103720

Orientation #**10**
phenylacetylene/ortho/phenylacetylene-tempo-orthoCH.freq.out
C -3.451624 -2.301647 0.165964
C -2.452213 -1.711955 -0.608729
C -2.378686 -0.327629 -0.724522
C -3.303050 0.491637 -0.049806
C -4.307634 -0.112030 0.730206
C -4.378540 -1.497448 0.831737
H -3.509609 -3.389198 0.253131
H -1.713802 -2.325392 -1.130587
H -1.583810 0.121166 -1.322544
H -5.027338 0.519891 1.254755
H -5.163165 -1.954650 1.439098
C -3.205945 1.910381 -0.146189
C 2.503417 -1.181294 -0.056150
C 3.812670 -0.581327 0.462194
C 3.618164 0.677119 1.283417
C 2.913195 1.723794 0.444172
C 1.540467 1.273421 -0.060937
H 4.344569 -1.358642 1.034077
H 3.042047 0.459738 2.199051
H 4.589783 1.058163 1.632036
H 2.781535 2.665284 1.001086
H 3.545960 1.970024 -0.426370
H 4.456763 -0.345685 -0.402807
C 1.720055 -1.877086 1.059116
H 0.713191 -2.139119 0.705059
H 2.228851 -2.804223 1.360269
H 1.612082 -1.254841 1.957244
C 2.798693 -2.181440 -1.165441
H 3.486354 -2.953935 -0.793796
H 1.882080 -2.667841 -1.517902
H 3.270122 -1.687407 -2.026059
C 0.506828 1.260896 1.067247
H 0.245643 2.289879 1.353394
H -0.414886 0.763239 0.735717

H 0.865213 0.748320 1.969702
C 1.065940 2.202753 -1.169623
H 0.048327 1.957482 -1.495145
H 1.067461 3.239626 -0.805088
H 1.727081 2.144250 -2.045098
N 1.654735 -0.099751 -0.648838
O 0.695920 -0.484168 -1.388041
C -3.104884 3.120054 -0.226208
H -3.026073 4.186270 -0.301700

Orientation #**11**
benzaldehyde/ring/benzaldehyde-tempo-ring.freq.out
C 3.207391 -1.841368 -1.208712
C 2.590012 -2.157245 0.003492
C 2.322058 -1.149450 0.925873
C 2.661722 0.177383 0.637367
C 3.282190 0.487820 -0.579809
C 3.555922 -0.519303 -1.499153
H 3.419788 -2.631546 -1.932969
H 1.823044 -1.373514 1.873362
C -2.386816 -0.669574 0.793566
C -3.672459 -0.344042 0.029836
C -3.444970 -0.041093 -1.436952
C -2.503954 1.139524 -1.566583
C -1.137951 0.902289 -0.919252
H -4.374284 -1.182508 0.166027
H -3.037239 -0.923717 -1.958907
H -4.402319 0.176505 -1.933594
H -2.339368 1.410562 -2.621760
H -2.969456 2.022212 -1.094500
H -4.149182 0.531269 0.503929
C -1.868245 -2.069338 0.457052
H -0.867723 -2.211675 0.889188
H -2.535204 -2.834619 0.879598
H -1.800526 -2.250829 -0.623898
C -2.638628 -0.570387 2.291940
H -3.463223 -1.240057 2.574021
H -1.747845 -0.850370 2.865468
H -2.915485 0.453638 2.577535
C -0.285304 -0.062521 -1.746032
H 0.064306 0.432883 -2.663822
H 0.599218 -0.369983 -1.171695
H -0.830347 -0.967391 -2.047050
C -0.399252 2.224577 -0.767476
H 0.625306 2.083088 -0.403876
H -0.350382 2.729849 -1.742250
H -0.916229 2.889644 -0.062585
N -1.329720 0.334786 0.453421
O -0.319342 0.358697 1.223591
H 2.317271 -3.192070 0.224629
H 4.040596 -0.279705 -2.448601
C 2.349053 1.249827 1.606639
O 2.556071 2.425256 1.408522

H 1.903070 0.887934 2.568942
H 3.535812 1.532683 -0.778812

Orientation #**12**
benzaldehyde/ortho/benzaldehyde-tempo-ortho.freq.out
C -4.299646 1.111616 -0.489090
C -3.786062 0.292274 -1.496433
C -2.888994 -0.721305 -1.172906
C -2.496646 -0.917480 0.156239
C -3.017712 -0.093366 1.163218
C -3.916501 0.917469 0.841041
H -5.004709 1.907442 -0.741657
H -2.458642 -1.357613 -1.951600
C 2.552241 -0.782308 -0.348480
C 3.737076 0.187354 -0.319256
C 3.509121 1.395433 0.566807
C 2.284313 2.152252 0.092867
C 1.008237 1.306518 0.101691
H 4.633129 -0.374503 -0.010332
H 3.391964 1.087533 1.619687
H 4.392491 2.051337 0.549874
H 2.105044 3.050893 0.704759
H 2.460684 2.512770 -0.935673
H 3.933508 0.532270 -1.349512
C 2.443822 -1.584007 0.950344
H 1.493191 -2.133642 0.990595
H 3.265518 -2.312462 1.010011
H 2.500990 -0.952477 1.846435
C 2.698187 -1.739738 -1.522607
H 3.665228 -2.257285 -1.455415
H 1.900573 -2.491295 -1.527168
H 2.662048 -1.201986 -2.479946
C 0.507383 1.066321 1.527993
H 0.054635 1.987086 1.924623
H -0.256061 0.277193 1.544184
H 1.307573 0.770071 2.218823
C -0.082110 1.996278 -0.705974
H -1.031832 1.450973 -0.646231
H -0.238630 3.011740 -0.315786
H 0.197874 2.074136 -1.765562
N 1.288191 -0.007769 -0.560082
O 0.288215 -0.655340 -0.994912
H -4.084831 0.450768 -2.535154
H -4.325335 1.559262 1.625129
C -1.520045 -1.974400 0.488728
O -1.041443 -2.138695 1.591099
H -1.252778 -2.644243 -0.368433
H -2.699143 -0.268768 2.194327

Orientation #**13**
benzaldehyde/meta2/benzaldehyde-tempo-meta2.freq.out

C 4.583876 -1.353880 0.131573
C 5.095737 -0.061860 -0.003673
C 4.221890 1.016194 -0.106575
C 2.835293 0.807943 -0.075386
C 2.326267 -0.494422 0.060640
C 3.202673 -1.567871 0.163603
H 5.268345 -2.202377 0.212333
H 4.607360 2.035299 -0.212908
C -1.934821 -0.084009 1.336409
C -2.448604 1.335731 1.088496
C -3.285303 1.459866 -0.168523
C -2.441257 1.065405 -1.363327
C -1.924602 -0.372922 -1.293310
H -3.010036 1.654431 1.981592
H -4.193292 0.835004 -0.102314
H -3.647228 2.492723 -0.283651
H -2.997247 1.180850 -2.307802
H -1.575855 1.745078 -1.423592
H -1.582389 2.011908 1.004199
C -3.047301 -1.006577 1.838781
H -2.687425 -2.044217 1.865710
H -3.350924 -0.720706 2.856237
H -3.943379 -0.971928 1.203925
C -0.800873 -0.051357 2.352165
H -1.154219 0.404292 3.287892
H -0.433682 -1.061270 2.570587
H 0.037662 0.548305 1.972511
C -3.032008 -1.385622 -1.593223
H -3.328031 -1.326935 -2.650601
H -2.670687 -2.403674 -1.393050
H -3.933408 -1.217802 -0.987644
C -0.781831 -0.557711 -2.282896
H -0.408957 -1.588957 -2.271822
H -1.128373 -0.319878 -3.298503
H 0.051465 0.115085 -2.037691
N -1.381159 -0.660213 0.071061
O -0.711941 -1.734890 0.191449
H 6.175607 0.100525 -0.027924
H 2.804458 -2.579652 0.269153
C 1.931078 1.961937 -0.188676
O 0.719772 1.907604 -0.178299
H 2.458272 2.949815 -0.291338
H 1.246134 -0.661215 0.085599

Orientation #**14**
benzaldehyde/ald/benzaldehyde-tempo-ald.freq.out
C -4.819217 -1.150293 -0.041915
C -3.534492 -1.694833 -0.050969
C -2.424577 -0.853778 -0.015281
C -2.605845 0.536458 0.029890
C -3.899808 1.079179 0.038080
C -5.002817 0.236073 0.002276
H -5.689311 -1.811041 -0.069866

H -1.408223 -1.267018 -0.023573
C 1.941079 -0.172394 -1.332868
C 2.225662 1.324081 -1.181677
C 3.017704 1.663622 0.065422
C 2.247730 1.209874 1.289569
C 1.964962 -0.294408 1.306991
H 2.741642 1.666411 -2.093184
H 4.016613 1.194893 0.034791
H 3.202190 2.746939 0.114003
H 2.779833 1.467366 2.219559
H 1.289535 1.757219 1.328485
H 1.267546 1.872264 -1.153227
C 3.193783 -0.947075 -1.748929
H 3.003617 -2.027344 -1.686074
H 3.459973 -0.706785 -2.788282
H 4.066823 -0.715461 -1.124479
C 0.850753 -0.395536 -2.372504
H 1.160882 0.042022 -3.331571
H 0.655683 -1.463963 -2.521377
H -0.093455 0.082413 -2.074477
C 3.224464 -1.102851 1.625694
H 3.512503 -0.957324 2.676827
H 3.031386 -2.173131 1.468804
H 4.084572 -0.815135 1.006472
C 0.893802 -0.613453 2.341069
H 0.699711 -1.691084 2.393032
H 1.222144 -0.267033 3.330766
H -0.054612 -0.109596 2.106758
N 1.439993 -0.726397 -0.030955
O 0.902684 -1.876918 -0.079115
H -3.398534 -2.777850 -0.086052
H -6.013160 0.651774 0.008412
C -1.422745 1.411239 0.068028
O -1.435281 2.621824 0.105717
H -0.450959 0.845405 0.057840
H -4.008214 2.166154 0.073020

Orientation #**15**
benzaldehyde/meta/benzaldehyde-tempo-meta.freq.out
C -2.339612 1.931636 -0.331472
C -1.893272 0.680944 -0.762966
C -2.721145 -0.428251 -0.602047
C -3.983259 -0.294459 -0.009598
C -4.419236 0.966592 0.423774
C -3.599030 2.075517 0.260617
H -1.698145 2.807360 -0.461193
H -2.392806 -1.416559 -0.940751
C 2.563235 1.167085 0.117398
C 2.951753 0.850818 1.562804
C 3.551733 -0.529066 1.737632
C 2.539644 -1.567207 1.298896
C 2.120546 -1.428281 -0.165755
H 3.637243 1.639271 1.912181

H 4.487906 -0.625793 1.161578
H 3.834722 -0.691963 2.788188
H 2.916137 -2.591351 1.450461
H 1.641706 -1.477198 1.934422
H 2.051553 0.928802 2.196729
C 3.792526 1.464205 -0.742534
H 3.498417 1.545222 -1.797696
H 4.251063 2.415936 -0.438480
H 4.562585 0.684930 -0.661522
C 1.615682 2.358565 0.086135
H 2.094676 3.229165 0.555323
H 1.343306 2.620850 -0.943411
H 0.691969 2.136841 0.639486
C 3.213802 -1.925155 -1.112775
H 3.323367 -3.015958 -1.031373
H 2.946750 -1.682423 -2.150166
H 4.193804 -1.477218 -0.898571
C 0.836025 -2.207996 -0.411651
H 0.527996 -2.141503 -1.462222
H 0.985900 -3.265991 -0.155415
H 0.018415 -1.817914 0.211216
N 1.831140 0.006846 -0.484722
O 1.276888 0.224098 -1.607628
H -0.904981 0.573665 -1.221220
H -3.935909 3.060365 0.592844
C -4.848902 -1.476338 0.157353
O -5.948151 -1.465377 0.660887
H -4.398529 -2.431442 -0.231240
H -5.407963 1.043577 0.882942

Orientation #**16**
benzaldehyde/ortho2/benzaldehyde-tempo-ortho2.freq.out
C 3.070878 -2.303412 -0.022540
C 4.119830 -1.766575 0.726851
C 4.291932 -0.386365 0.779685
C 3.417081 0.461280 0.086203
C 2.367261 -0.086242 -0.666405
C 2.195613 -1.464179 -0.718357
H 2.934936 -3.387260 -0.065094
H 5.108934 0.050231 1.363221
C -2.300914 -1.250965 -0.054797
C -2.873113 -1.064546 1.351696
C -3.449382 0.316047 1.589147
C -2.361232 1.350420 1.388230
C -1.749642 1.332706 -0.014161
H -3.623910 -1.852622 1.522942
H -4.300125 0.506060 0.912526
H -3.861868 0.387647 2.606704
H -2.729343 2.369583 1.586731
H -1.558571 1.169495 2.124087
H -2.069153 -1.245677 2.086177
C -3.409941 -1.390964 -1.099400
H -2.976042 -1.362577 -2.108279

H -3.930897 -2.351436 -0.976487
H -4.163965 -0.595582 -1.029332
C -1.416862 -2.490161 -0.090044
H -1.989801 -3.363993 0.250686
H -1.052571 -2.689239 -1.105028
H -0.545873 -2.372003 0.570084
C -2.696339 1.942014 -1.049601
H -2.782932 3.026740 -0.893208
H -2.299899 1.773253 -2.060087
H -3.709281 1.519584 -1.003277
C -0.438429 2.104505 -0.010185
H -0.003128 2.177964 -1.013394
H -0.607391 3.124619 0.361597
H 0.302143 1.625141 0.645103
N -1.440548 -0.078802 -0.416069
O -0.716066 -0.220960 -1.450399
H 4.801187 -2.427051 1.267938
H 1.368415 -1.875107 -1.300602
C 3.602856 1.921651 0.159973
O 2.888226 2.743237 -0.366783
H 4.492911 2.238126 0.772088
H 1.685205 0.576217 -1.202923

Orientation #**17**
benzaldehyde/para/benzaldehyde-tempo-para.freq.out
C -1.821175 -0.331722 -1.008812
C -2.304024 0.977987 -0.979974
C -3.558104 1.243018 -0.436382
C -4.336425 0.199922 0.082047
C -3.848554 -1.115081 0.048816
C -2.597251 -1.376670 -0.495139
H -0.831111 -0.525784 -1.430995
H -3.949495 2.265171 -0.408356
C 2.394123 1.338379 0.098150
C 2.757744 1.178562 1.575312
C 3.582176 -0.059606 1.861732
C 2.795620 -1.285976 1.447085
C 2.429723 -1.302836 -0.038441
H 3.275140 2.095022 1.900966
H 4.549563 -0.017260 1.332617
H 3.834175 -0.111713 2.931350
H 3.342385 -2.214954 1.674490
H 1.865831 -1.328034 2.040375
H 1.826200 1.129801 2.165378
C 3.596935 1.791028 -0.731278
H 3.349353 1.748818 -1.800665
H 3.864255 2.827616 -0.481048
H 4.487235 1.170451 -0.561499
C 1.264552 2.348250 -0.050541
H 1.561452 3.307323 0.396220
H 1.020358 2.515246 -1.106300
H 0.355840 1.998629 0.459655
C 3.639488 -1.635861 -0.912877

H 3.938396 -2.683876 -0.768372
H 3.384174 -1.494249 -1.971833
H 4.512234 -1.009138 -0.684828
C 1.326906 -2.323680 -0.283486
H 1.065667 -2.380136 -1.347391
H 1.659894 -3.317340 0.047028
H 0.420479 -2.062975 0.281039
N 1.895920 0.035864 -0.450240
O 1.352301 0.090272 -1.597781
H -1.692799 1.787032 -1.386690
H -2.216591 -2.401008 -0.525349
C -5.662289 0.486142 0.659323
O -6.405254 -0.340807 1.135020
H -5.949754 1.573621 0.627320
H -4.477366 -1.910165 0.457441

Orientation #**18**
nitrobenzene/tshape/nitrobenzene-tempo-tshape.freq.out
C -2.930927 -2.467273 0.000046
C -1.685357 -1.837405 0.000007
C -1.615523 -0.446728 -0.000064
C -2.796399 0.289479 -0.000066
C -4.048730 -0.322006 0.000000
C -4.106893 -1.712323 0.000040
H -2.987300 -3.558061 0.000079
H -0.754967 -2.412491 0.000023
H -0.653973 0.068302 -0.000165
H -4.945698 0.297074 0.000015
H -5.078026 -2.211421 0.000063
C 2.037971 -0.098693 -1.318630
C 1.695954 1.390870 -1.239067
C 2.241459 2.072870 -0.000172
C 1.695789 1.391110 1.238778
C 2.037782 -0.098435 1.318707
H 2.061230 1.875674 -2.158654
H 3.345376 2.061248 -0.000112
H 1.956603 3.135417 -0.000270
H 2.060909 1.876126 2.158316
H 0.597997 1.508034 1.250157
H 0.598170 1.507808 -1.250621
C 3.510843 -0.327425 -1.665895
H 3.766281 -1.388649 -1.539568
H 3.697287 -0.054826 -2.714508
H 4.195477 0.267001 -1.047447
C 1.172825 -0.776416 -2.372117
H 1.330246 -0.288938 -3.344106
H 1.422129 -1.839453 -2.469016
H 0.104588 -0.699130 -2.126901
C 3.510581 -0.327118 1.666304
H 3.696819 -0.054298 2.714896
H 3.766034 -1.388370 1.540247
H 4.195324 0.267184 1.047858
C 1.172445 -0.775897 2.372208

H 1.421719 -1.838917 2.469393
H 1.329707 -0.288201 3.344111
H 0.104254 -0.698662 2.126783
N 1.736846 -0.749817 0.000088
O 1.664149 -2.019048 0.000217
N -2.719649 1.760279 -0.000136
O -1.608051 2.270597 -0.000428
O -3.768783 2.380534 0.000122

Orientation #**19**
nitrobenzene/bite-orthometa/nitrobenzene-tempo-bite-orthometa.freq.out
C -4.372294 1.751384 -0.000068
C -2.984879 1.899252 -0.000316
C -2.155733 0.780231 -0.000342
C -2.751072 -0.479926 -0.000093
C -4.134991 -0.652073 0.000158
C -4.945955 0.477646 0.000165
H -5.014172 2.635261 -0.000040
H -2.533416 2.893376 -0.000477
H -1.069572 0.894544 -0.000556
H -4.546821 -1.661083 0.000350
H -6.031876 0.364382 0.000357
C 2.072814 0.251102 1.322171
C 2.499815 -1.215582 1.235003
C 3.319671 -1.531069 0.000394
C 2.500223 -1.215784 -1.234530
C 2.073144 0.250853 -1.322078
H 3.046859 -1.465709 2.158297
H 4.266927 -0.963942 0.000515
H 3.612537 -2.591677 0.000521
H 3.047575 -1.466031 -2.157608
H 1.594710 -1.844651 -1.228972
H 1.594260 -1.844407 1.229249
C 3.241827 1.157863 1.713628
H 2.945218 2.211668 1.620483
H 3.533906 0.974229 2.757605
H 4.130920 0.996416 1.088981
C 0.950902 0.401918 2.340670
H 1.289270 0.035038 3.319781
H 0.646411 1.450098 2.445592
H 0.073233 -0.186547 2.039725
C 3.242195 1.157593 -1.713455
H 3.534516 0.973766 -2.757330
H 2.945542 2.211410 -1.620565
H 4.131143 0.996282 -1.088567
C 0.951476 0.401396 -2.340893
H 0.647055 1.449557 -2.446220
H 1.290071 0.034213 -3.319811
H 0.073711 -0.186936 -2.039980
N 1.540944 0.709048 -0.000064
O 0.910296 1.813502 -0.000267
N -1.897774 -1.679124 -0.000064
O -0.684426 -1.511341 -0.000181

O -2.445003 -2.768104 0.000081

Orientation #**20**
nitrobenzene/ortho/nitrobenzene-tempo-orthoCH.freq.out
C -3.252988 -2.101629 0.734961
C -3.085835 -1.791026 -0.617072
C -2.694026 -0.512308 -0.998427
C -2.464790 0.437945 -0.007126
C -2.619420 0.149049 1.345364
C -3.020931 -1.133461 1.712637
H -3.565315 -3.106555 1.027774
H -3.260813 -2.553327 -1.379360
H -2.538762 -0.234252 -2.040500
H -2.428650 0.928814 2.083149
H -3.155461 -1.376798 2.768617
C 1.657339 -1.392437 -0.460388
C 3.157906 -1.571367 -0.219356
C 3.712855 -0.651594 0.849629
C 3.463939 0.790369 0.454331
C 1.980883 1.122726 0.278427
H 3.343426 -2.630379 0.022099
H 3.254385 -0.871411 1.829104
H 4.790215 -0.831085 0.983239
H 3.887047 1.489906 1.192707
H 3.987252 0.996494 -0.495603
H 3.690767 -1.377701 -1.166535
C 0.822778 -2.007145 0.665231
H -0.229774 -1.709772 0.552560
H 0.873681 -3.104908 0.621983
H 1.159830 -1.700258 1.664444
C 1.264620 -2.035999 -1.783470
H 1.568895 -3.092091 -1.784868
H 0.181523 -1.984020 -1.946075
H 1.757714 -1.535468 -2.627974
C 1.260764 1.205298 1.625805
H 1.600838 2.092712 2.178800
H 0.179029 1.308297 1.466871
H 1.441581 0.329387 2.263310
C 1.820826 2.444888 -0.459933
H 0.767390 2.739931 -0.536181
H 2.362664 3.233750 0.080206
H 2.232743 2.380500 -1.476379
N 1.333333 0.065046 -0.561063
O 0.198145 0.350966 -1.050468
N -2.054218 1.793602 -0.406017
O -1.676004 2.548610 0.479803
O -2.131833 2.085156 -1.583774

Orientation #**21**
nitrobenzene/para/nitrobenzene-tempo-paraCH.freq.out
C 1.806927 1.056703 -0.796680

C 1.903816 0.368550 0.415190
C 3.134497 -0.115143 0.848255
C 4.255242 0.102832 0.049371
C 4.181271 0.787180 -1.161497
C 2.942773 1.264981 -1.581163
H 0.827781 1.422940 -1.113761
H 1.000483 0.219960 1.010495
H 3.250072 -0.655611 1.787751
H 5.089105 0.930909 -1.747209
H 2.865200 1.804267 -2.527634
C -3.561773 0.951924 0.482864
C -4.810798 0.369723 -0.181742
C -4.797624 -1.142507 -0.270748
C -3.593583 -1.584410 -1.077119
C -2.259740 -1.142111 -0.471947
H -5.692716 0.735751 0.367693
H -4.780744 -1.593580 0.736106
H -5.724531 -1.505268 -0.739274
H -3.567319 -2.678857 -1.199641
H -3.674326 -1.166788 -2.095730
H -4.894441 0.785211 -1.200970
C -3.578459 0.737747 1.997409
H -2.608273 1.023902 2.425921
H -4.354954 1.362227 2.461709
H -3.782710 -0.304435 2.277389
C -3.461081 2.441033 0.182661
H -4.371607 2.950810 0.526762
H -2.596960 2.890249 0.684990
H -3.356759 2.619153 -0.896500
C -1.901602 -1.972156 0.762109
H -1.657026 -3.002875 0.468388
H -1.021855 -1.544447 1.262481
H -2.719097 -2.022689 1.493622
C -1.151951 -1.274712 -1.508096
H -0.169449 -1.043281 -1.079541
H -1.126909 -2.303540 -1.893196
H -1.321077 -0.596699 -2.356413
N -2.334993 0.302083 -0.081634
O -1.230973 0.868571 0.190851
N 5.562361 -0.407073 0.500031
O 5.595999 -1.014738 1.557726
O 6.528606 -0.190108 -0.213004

Orientation #**22**
nitrobenzene/stackedtshape/nitrobenzene-tempo-stackedtshape.freq.out
C -2.531439 2.652338 -0.024409
C -1.764271 1.762649 0.728591
C -2.093046 0.410076 0.761710
C -3.193204 -0.025156 0.027908
C -3.972894 0.846278 -0.731065
C -3.633084 2.195141 -0.752213
H -2.269741 3.712996 -0.046459
H -0.893943 2.103378 1.291866

H -1.496584 -0.295398 1.339057
H -4.823726 0.452826 -1.286954
H -4.231034 2.894308 -1.340461
C 1.825759 -1.391086 0.000472
C 2.430225 -1.475806 -1.402469
C 3.626606 -0.568376 -1.601280
C 3.207250 0.867716 -1.364180
C 2.657616 1.116515 0.041724
H 2.683394 -2.529292 -1.601223
H 4.449393 -0.852098 -0.922935
H 4.030477 -0.685034 -2.618007
H 4.041326 1.567295 -1.534036
H 2.429610 1.137246 -2.099855
H 1.652504 -1.206904 -2.138196
C 2.702375 -2.099248 1.034768
H 2.330536 -1.886976 2.046386
H 2.668686 -3.187045 0.879745
H 3.755385 -1.790964 0.984137
C 0.438501 -2.015928 0.000830
H 0.493967 -3.045607 -0.378633
H 0.005725 -2.049518 1.007205
H -0.249792 -1.454365 -0.646898
C 3.773531 1.132519 1.087873
H 4.397543 2.029519 0.965934
H 3.338531 1.151798 2.096452
H 4.435153 0.258839 1.017332
C 1.915784 2.445862 0.075274
H 1.573436 2.684662 1.089407
H 2.582715 3.250885 -0.263653
H 1.039588 2.425179 -0.588345
N 1.672314 0.046100 0.401113
O 0.963388 0.267621 1.432580
N -3.545408 -1.456493 0.047016
O -2.838813 -2.203729 0.704312
O -4.520794 -1.805634 -0.598139

Orientation #**23**
nitrobenzene/meta/nitrobenzene-tempo-metaCH.freq.out
C 2.562620 2.429222 -0.205845
C 1.840552 1.237120 -0.301145
C 2.502020 0.018527 -0.168616
C 3.876214 0.019103 0.058451
C 4.610854 1.198180 0.156150
C 3.940191 2.410394 0.021058
H 2.042212 3.383743 -0.310806
H 0.759701 1.258437 -0.477047
H 1.978092 -0.935735 -0.236931
H 5.684675 1.143722 0.334362
H 4.497309 3.346759 0.093557
C -3.613823 0.808862 0.235120
C -4.669840 -0.296086 0.169285
C -4.136966 -1.663047 0.546734
C -3.014344 -2.038637 -0.398663

C	-1.841607	-1.056087	-0.374424
H	-5.515393	0.000674	0.810087
H	-3.787302	-1.671594	1.593211
H	-4.940993	-2.412490	0.500318
H	-2.621164	-3.044558	-0.181885
H	-3.415230	-2.084394	-1.426027
H	-5.069476	-0.341631	-0.858651
C	-3.290059	1.192325	1.680181
H	-2.427091	1.871763	1.701521
H	-4.145051	1.709919	2.138077
H	-3.055844	0.322565	2.308649
C	-4.101797	2.038110	-0.519020
H	-5.058352	2.376724	-0.097375
H	-3.378202	2.858291	-0.451269
H	-4.257637	1.811191	-1.582661
C	-1.000265	-1.222714	0.892752
H	-0.442786	-2.170047	0.860534
H	-0.273977	-0.402421	0.978677
H	-1.608420	-1.230310	1.807253
C	-0.967745	-1.270516	-1.602874
H	-0.097144	-0.604516	-1.601196
H	-0.610620	-2.309472	-1.628769
H	-1.531960	-1.079259	-2.525940
N	-2.353572	0.350494	-0.433184
O	-1.492300	1.254504	-0.668789
N	4.575613	-1.270543	0.200999
O	5.778184	-1.240323	0.404156
O	3.907159	-2.287373	0.107476
